# The LCK-14-3-3ζ-TRPM8 axis regulates TRPM8 function/assembly and promotes pancreatic cancer malignancy

**DOI:** 10.1038/s41419-022-04977-5

**Published:** 2022-06-04

**Authors:** Yuan Huang, Shi Li, Qinfeng Liu, Zhijie Wang, Shunyao Li, Lei Liu, Weiwei Zhao, Kai Wang, Rui Zhang, Longfei Wang, Ming Wang, Declan William Ali, Marek Michalak, Xing-Zhen Chen, Cefan Zhou, Jingfeng Tang

**Affiliations:** 1 National “111” Center for Cellular Regulation and Molecular Pharmaceutics, Key Laboratory of Fermentation Engineering (Ministry of Education), Hubei University of Technology, Wuhan, China; 2 Department of Obstetrics and Gynecology, Tongji Hospital, Tongji Medical College, Huazhong University of Science and Technology, 430030 Wuhan, China; 3 Children’s Hospital Affiliated to Zhengzhou University, Henan Key Laboratory of Children’s Genetics and Metabolic Diseases, Henan Children’s Hospital, Zhengzhou Children’s Hospital, Zhengzhou, 450018 China; 4 Department of Clinical Laboratory, Renmin Hospital of Wuhan University, Wuhan, China; 5 Department of Biological Sciences, University of Alberta, Edmonton, AB T6G 2H7 Canada; 6 Department of Biochemistry, University of Alberta, Edmonton, AB T6G 2H7 Canada; 7 Membrane Protein Disease Research Group, Department of Physiology, Faculty of Medicine and Dentistry of Alberta, Edmonton, AB T6G 2H7 Canada

**Keywords:** Phosphorylation, Paediatric cancer

## Abstract

Transient receptor potential melastatin 8 (TRPM8) functions as a Ca^2+^-permeable channel in the plasma membrane (PM). Dysfunction of TRPM8 is associated with human pancreatic cancer and several other diseases in clinical patients, but the underlying mechanisms are unclear. Here, we found that lymphocyte-specific protein tyrosine kinase (LCK) directly interacts with TRPM8 and potentiates TRPM8 phosphorylation at Y1022. LCK positively regulated channel function characterized by increased TRPM8 current densities by enhancing TRPM8 multimerization. Furthermore, 14-3-3ζ interacted with TRPM8 and positively modulated channel multimerization. LCK significantly enhanced the binding of 14-3-3ζ and TRPM8, whereas mutant TRPM8-Y1022F impaired TRPM8 multimerization and the binding of TRPM8 and 14-3-3ζ. Knockdown of 14-3-3ζ impaired the regulation of TRPM8 multimerization by LCK. In addition, TRPM8 phosphotyrosine at Y1022 feedback regulated LCK activity by inhibiting Tyr505 phosphorylation and modulating LCK ubiquitination. Finally, we revealed the importance of TRPM8 phosphorylation at Y1022 in the proliferation, migration, and tumorigenesis of pancreatic cancer cells. Our findings demonstrate that the LCK-14-3-3ζ-TRPM8 axis for regulates TRPM8 assembly, channel function, and LCK activity and maybe provide potential therapeutic targets for pancreatic cancer.

## Introduction

Pancreatic cancer is an aggressive malignancy with high mortality, and only 5–7% of patients live longer than five years after diagnosis [1]. Although recent advances in radiotherapy and chemotherapy have shown promising results, the overall prognosis and survival rates of pancreatic cancer patients are limited [2]. Therefore, understanding the biology of pancreatic cancer and identifying putative therapeutic targets in clinical treatment are urgently needed. Transient receptor potential melastatin 8 (TRPM8), the first identified prostate-specific gene, was functionally characterized as a cold receptor due to its activation by cold temperature and substances that mimic cold sensation such as menthol and icilin, and plays a central role in thermosensation [3]. Recently, several studies revealed that TRPM8 exhibits aberrant expression and contributes to the development and progression of pancreatic cancer [4, 5]. Identification of the mechanisms by which TRPM8 mediates its biological functions is expected to develop into a molecular biomarker and therapeutic target in pancreatic cancer.

TRPM8 belongs to the TRP channel family and functions as a nonselective, voltage-gated, and Ca^2+^-permeable channel that must be correctly expressed and assembled in the plasma membrane (PM). Previous studies showed that four monomers were assembled to form a homologous tetramer of functional TRPM8 channels [6–8]. Although the C-terminal coiled coil (K1066-K1104) of TRPM8 has been implicated in channel multimerization [6–8], the mechanism remains obscure. In addition, several molecules, such as PIP2, PKA, PKC, TRP channel-associated factors (TACF1 and TACF2), tripartite motif‐containing 4 (TRIM4), and ubiquitin-like modifier activating enzyme 1 (UBA1), modulate TRPM8 channel expression and activity in the PM [9–14]. Earlier studies revealed that 4-amino-5-(4-chlorophenyl)-7-(dimethylethyl) pyrazolo[3,4-d] pyrimidine (PP2, a selective Src family tyrosine kinase inhibitor) inhibited TRPM8 function in SH-SY5Y and HEK293T cells and TRPM8 is phosphotyrosined by Src, a membrane of nonreceptor Src family kinases, and partly by a representative of receptor PTKs, TrkA, without identifying the exact site(s) [15, 16]. Thus, the mechanism of TRPM8 regulation by tyrosine kinases needs to be further investigated.

Lymphocyte-specific protein tyrosine kinase (LCK), which functions as a Src-related nonreceptor protein tyrosine kinase, has emerged as one of the key molecules regulating T-cell functions [17, 18]. Dysregulated LCK, similar to other Src kinases, is associated with various disease conditions such as cancers, asthma, and diabetes [19]. The mechanistic insights into the regulation of LCK activity are sophisticated. Currently, LCK activity is predominantly regulated via reversible and dynamic phosphorylation of two tyrosine residues, one within the “activation loop” of the catalytic domain Y394 and the other at the carboxy-terminus (C-terminus) of the protein Y505 [20–22]. Blocking phosphorylation on Tyr394 (Y394F) largely reduced LCK activity, whereas inhibition of Tyr505 phosphorylation (Y505F) stimulated LCK activity [20–22]. In addition to phosphorylation, ubiquitination is also involved in regulating LCK activity [23–25]. For example, heat shock protein 90 (Hsp90) prevents the active form Y505F of mutant LCK from being targeted for degradation by ubiquitination [23]. 14-3-3 is a family of small acidic proteins that are widely expressed in many organisms and tissues and consists of seven highly conserved ~30 kDa isoforms (β, ε, γ, η, σ, τ, ζ) [26]. By forming dimers, 14-3-3 predominantly binds phosphorylated proteins to modulate their targets at various levels, such as subcellular localization, stability, multimerization, phosphorylation, biological activity, or dynamic interactions [27, 28]. TRPM7, which belongs to the TRP channel family, binds to 14-3-3 to modulate channel cellular localization that requires autophosphorylation at S1403 [29]. Apart from TRPM7, 14-3-3 which is involved in the regulation of other TRP channel functions is limited. In addition, there is no report that 14-3-3 is involved in the regulation of LCK on target proteins.

In this study, we employed various biological approaches to identify and unveil the mechanisms by which the LCK-14-3-3ζ-TRPM8 axis regulates TRPM8 assembly, channel function, and LCK activity and highlighted the importance of TRPM8 phosphotyrosine at Y1022 on the pancreatic cancer cells, which may be a potential therapeutic target for pancreatic cancer.

## Results

### LCK-TRPM8 interaction for positively modulates TRPM8 phosphotyrosine

We have previously reported a GST pull-down assay in combination with mass spectrometric (MS) analysis to screen candidate proteins from an ~60 kD bands that bind to the C-terminus of TRPM8 (M8C, amino acid 980-1104). The Ub-ligase E3 for TRIM4 was identified as a novel partner of TRPM8 in our earlier report [10]. Within the same screen, we also identified peptides for BLK, LCK, and LYN, belonging to the Src family kinases, which were found in a similar size of ~60 kD band using co-immunoprecipitation (Co-IP) and MS assays in MCF7 cells (Fig. S1A–D).

**Fig. 1:**
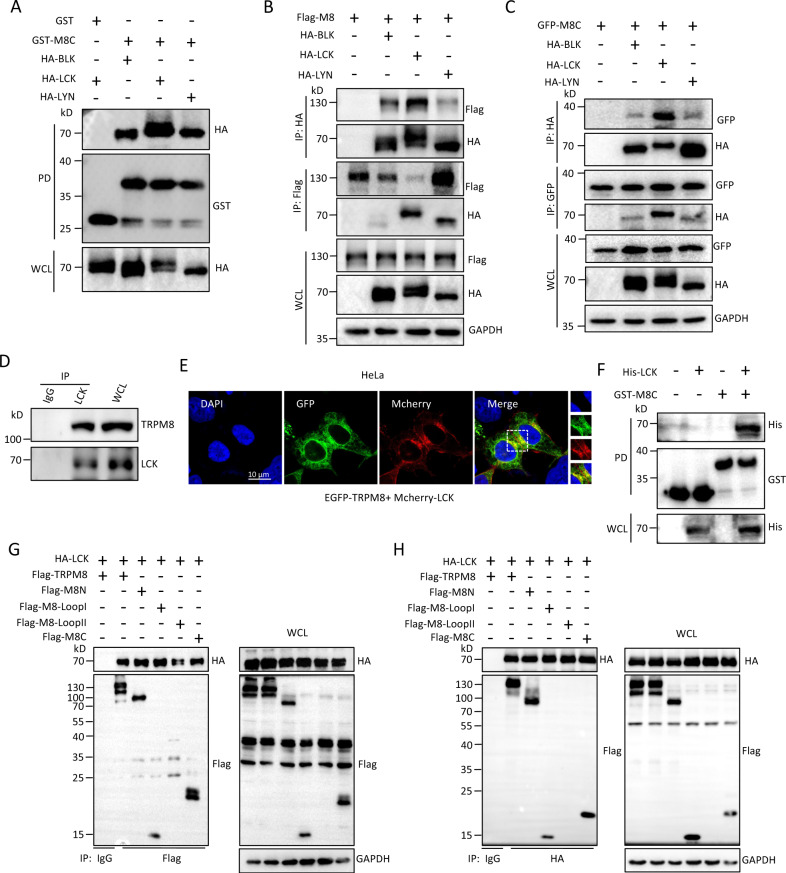
Src family kinases for BLK, LCK, and LYN interaction with TRPM8. **A** GST pull-down assays to assess the interaction between purified GST-tagged C-terminus of TRPM8 (GST-M8C) expressing in E. coli BL21 bacteria and different HA-tagged Src family kinases expressing in HEK293T cells. Western blotting (WB) was performed using the indicated antibodies. **B**–**D** Co-immunoprecipitation (Co-IP) assays. **B** HeLa cells were transfected with the indicated constructs along with Flag-tagged full-length TRPM8 (Flag-TRPM8). Immunoprecipitation (IP) was performed with an anti-HA or anti-Flag antibody, and the samples were analyzed by immunoblotting with the indicated antibodies. **C** Similar Co-IP in (**B**) but with protein extracts from HeLa cells co-transfected with the indicated constructs along with GFP-tagged C-terminus of TRPM8 (GFP-M8C). **D** Co-IP as in (**B**) and (**C**) but with protein extracts from native PANC-1 cells. **E** Representative confocal imaging of co-localization of mcherry-LCK and GFP-TRPM8 in HeLa cells. Overlay images show co-localization of green signals (TRPM8) and red signals (LCK), which generated yellow signals in HeLa. Nuclei were stained with DAPI (blue). Scale bars, 10 µm. **F** Assay of the interaction in vitro between purified His-LCK fusion and GST-tagged C-terminus of TRPM8 (GST-M8C) from E. coli bacteria. **G**, **H** HEK293T cells co-expressing HA-LCK constructs with a series of mutant Flag-tagged cytoplasmic domain of TRPM8 were harvested for Co-IP assays. All studies were repeated at least three times. GFP, green fluorescent protein. All studies were repeated at least three times.

To further document the interaction between TRPM8 and these Src family kinases, we performed an in vitro GST pull-down assay. The results indicated that purified GST-M8C more efficiently pulled down HA-tagged LCK compared with HA-tagged BLK and LYN from HEK293T cell lysate (Fig. 1A). Moreover, we also confirmed that the BLK, LCK, and LYN kinases interacted with full-length TRPM8 and that LCK-TRPM8 binding was strongest (Fig. 1B). Consistent with the above assays, reciprocal Co-IP experiments showed that BLK, LCK, and LYN were associated with the C-terminus of TRPM8 in a protein complex, and the strongest interaction of C-termini of TRPM8 and LCK was clearly observed (Fig. 1C).

Src family kinases are involved in the phosphotyrosine of target proteins. PP2, a widely used compound to block the activity of Src family kinases, was first used to determine the effect of Src family kinases on TRPM8 phosphotyrosine. A 24-h exposure of transfected HeLa cells to different concentrations of PP2 (0, 2.5, 10, 20 μM) resulted in a dose-dependent reduction in TRPM8 phosphotyrosine (Fig. S2, A, B). In contrast, the reduction was counteracted by co-application of 1 mM orthovanadate, a protein tyrosine phosphatase inhibitor (Fig. S2, C, D). Notably, orthovanadate itself significantly enhanced the basal phosphotyrosine of TRPM8 (Fig. S2, C, D). Moreover, we confirmed that only LCK overexpression, but not BLK or LYN overexperssion, resulted in a significant increase in TRPM8 phosphotyrosine by ~2.19-fold (Fig. S2, E, F), which is consistent with the strongest interaction between LCK and TRPM8 (Fig. 1A–C). These data suggested that LCK, as a Src family kinase, is a potent positive regulator in the regulation of TRPM8 phosphotyrosine via the LCK-TRPM8 interaction.

We next performed a Co-IP assay to detect the endogenous interaction of TRPM8 and LCK in native cells. The results showed that LCK effectively co-precipitated with TRPM8 in PANC-1 cells (Fig. 1D). In addition, TRPM8 colocalized with LCK in the cytoplasm as shown by confocal microscopy (Fig. 1E) and purified His-LCK successfully pulled down purified GST-M8C but not GST alone in BL21 bacteria as shown by a protein-protein interaction assay in vitro (Fig. 1F). Moreover, cells expressing with increasing amounts of LCK markedly enhanced TRPM8 phosphotyrosine in a dose-dependent manner (Fig. S2, G, H), and LCK knockdown significantly decreased TRPM8 phosphotyrosine to ~37% (Fig. S2, I, J). Meanwhile, LCK overexpression markedly enhanced the phosphorylation of the C- terminus of TRPM8 by ~2.87-fold in HEK293T cells (Fig. S2, K, L), and the immunoprecipitated LCK from transfected HEK293T cells effectively phosphorylated M8C proteins purified from BL21 bacteria by kinase assay in vitro (Fig. S2M). We also determined which domain(s) of TRPM8 in the cytoplasm that binds with LCK. Reciprocal Co-IP assays showed that LCK binds with more than one cytosolic domain of TRPM8 cytosolic (Fig. 1G, H). Together, these data strongly suggested that LCK binds to TRPM8 and positively regulates TRPM8 phosphotyrosine.

### LCK increases TRPM8 channel activities

**Fig. 2:**
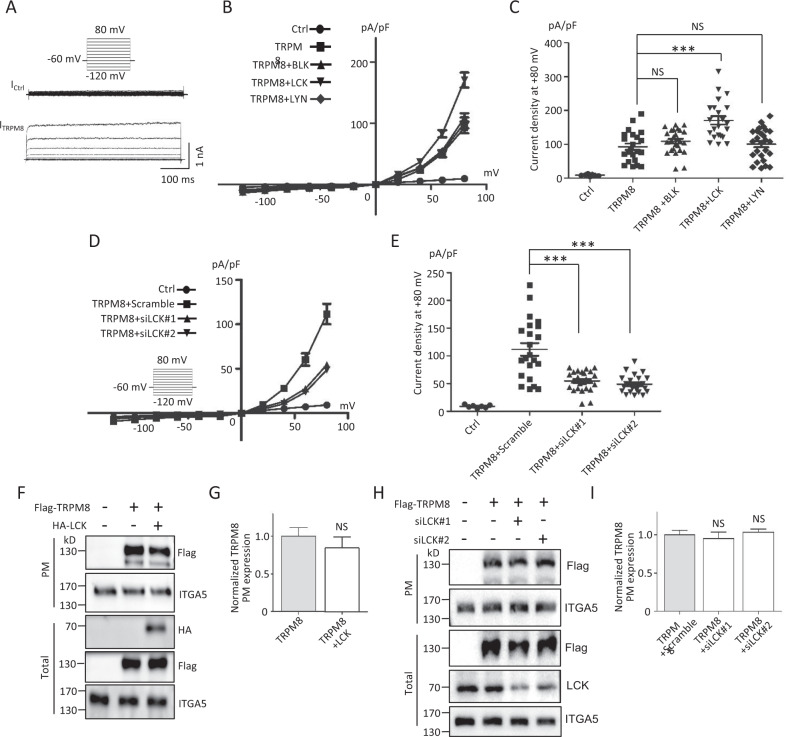
Functional effects of LCK on TRPM8 in HEK293T cells. **A**–**E** Electrophysiological analysis of LCK on whole-cell TRPM8 currents (I_TRPM8_) by patch-clamping experiments. **A**–**C** Expression constructs for Flag-TRPM8 and pEGFP-N1 were co-transfected with HA-BLK, HA-LCK, HA-LYN, or control vector into HEK293T cells. EGFP-positive cells were selected for recording I_TRPM8_ (*n* = 5~20 cells per group). The voltage clamp protocol is shown in the *inset* of figure. **A** Representative imaging of I_TRPM8_. **B** The relationship of average I_TRPM8_ densities (I_TRPM8_ normalized to cell capacitance) and voltage. **C** Quantification of peak I_TRPM8_ density on +80 mV as in (**B**). **D**, **E** Similar I_TRPM8_ recordings as in (**A**) but HEK293T cells co-expressing Flag-TRPM8 and pEGFP-N1 with siLCK or negative scramble siRNAs. (*n* = 5~20 cells per group). **D** The relationship of average I_TRPM8_ densities and voltage. **E** Quantification of peak I_TRPM8_ density on +80 mV as in (**D**). **F**–**I** Cell‐surface biotinylation assays for detecting TRPM8 PM expression. **F** Representative WB images of TRPM8 on the PM and total lysates from HEK293T cells co-transfected with or without HA-LCK. **G** Quantification of PM protein expression levels of TRPM8 in (**F**). **H**, **I** Similar experiments in (**F**) and (**G**) but cells co-expressing with siLCK or negative scramble siRNAs. ITGA5 (Intergrin α5) was used as a loading control for PM proteins. ***P < 0.001, NS not significant. Data are presented as mean ± SEM. All studies were repeated at least three times.

We next employed patch‐clamp electrophysiology in HEK293 cells recording whole-cell TRPM8-mediated cation currents (I_TRPM8_) (Fig. 2A) to characterize the functional role of BLK, LCK and LYN in the TRPM8 channel. Compared with the control, overexpressed LCK increased I_TRPM8_ densities across at depolarization and markedly increased I_TRPM8_ densities by 1.8-fold at +80 mV, whereas BLK and LYN were rarely affected (Fig. 2B, C), which was consistent with the above result that only LCK markedly enhanced TRPM8 phosphorylation (Fig. S2E, F). LCK knockdown reduced I_TRPM8_ densities across at depolarization and markedly decreased I_TRPM8_ densities at +80 mV to ~46% (Fig. 2D, E).

To assess the molecular mechanism by which LCK enhances TRPM8 expression on the PM, we extracted TRPM8 proteins from the PM of transfected HEK293T cells. The results showed that overexpression of LCK and LCK knockdown rarely affected the PM and total expression of TRPM8 (Fig. 2F–I), suggesting that LCK modulated TRPM8 channel function likely by regulating the biophysical properties of the TRPM8 channel but not PM TRPM8 trafficking. These data together indicated that LCK acts as a positive regulator of TRPM8-mediated currents.

### LCK affects the multimerization but not intramolecular N-C binding of TRPM8

**Fig. 3:**
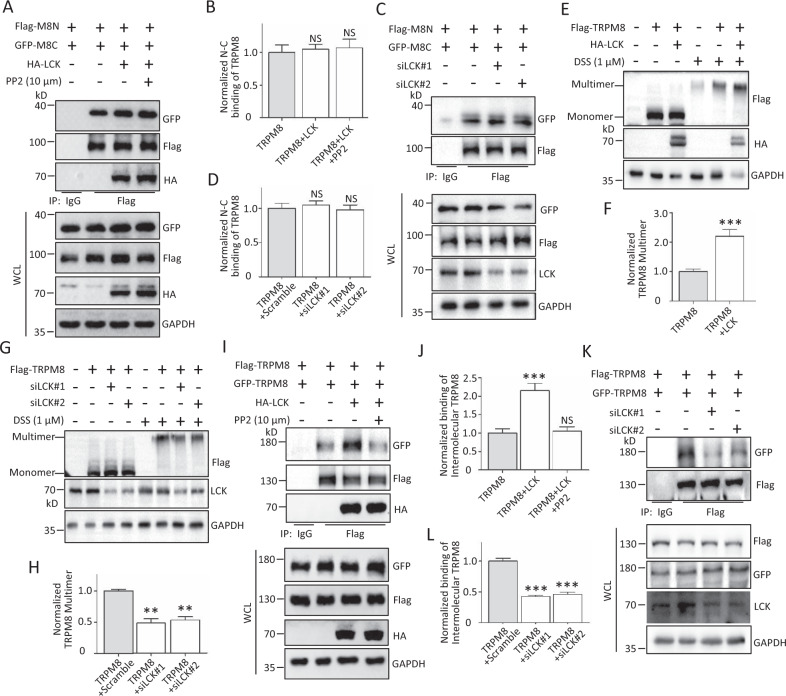
The multimerization, but not intracellular N-C binding of TRPM8, regulated by LCK. **A**–**D** The effect of LCK on the intracellular N-C binding of TRPM8. **A**, **B** Expression constructs for Flag-tagged N-terminus of TRPM8 (Flag-M8N) and GFP-M8C were co-transfected with HA-LCK or control vector into HEK293T cells, before harvest for treatment with 10 μM PP2 for 24 h. The cells were then harvested for IP with an anti-Flag antibody and WB assay with the indicated antibodies to determine the intracellular N-C binding of TRPM8. **C**, **D** Similar experiments in (**A**) and (**B**) but cells co-expressing Flag-M8N and GFP-M8C along with siLCK. **E**–**L** The effect of LCK on the multimerization of TRPM8. **E**, **F** Expression constructs for Flag-TRPM8 were co-transfected with HA-LCK or control vector into PANC-1 cells, before harvest for treatment with 1 μM DSS for 30 min, a crosslinking agent. The cell lysates were subjected to WB assay with the indicated antibodies to detect the level of TRPM8 multimerization. **G**, **H** Similar experiments in (**E**) and (**F**) but cells co-expressing Flag-TRPM8 along with siLCK. **I**, **J** Flag-TRPM8 and GFP-TRPM8 were co-transfected with or without HA-LCK into AsPC-1 cells. The cells were then harvested for IP with an anti-Flag antibody and WB assay with the indicated antibodies to determine the binding of intermolecular TRPM8. **K**, **L** Similar experiments in (**I**) and (**J**) but cells co-expressing Flag-TRPM8 and GFP-TRPM8 along with siLCK. **P < 0.01, ***P < 0.001, NS not significant. Data are presented as mean ± SEM. All studies were repeated at least three times.

Due to the LCK interaction with the N-terminus and C-terminus of TRPM8 (Fig. 1G, H) and the importance of the intramolecular N-C binding for the activation of TRPM8 function [12, 13], we investigated whether LCK affected the intramolecular N-C binding of TRPM8. The results showed that overexpressed LCK rarely affected the intramolecular N-C binding of TRPM8 with or without PP2 (Fig. 3A, B). Moreover, the intramolecular N-C binding of TRPM8 was not altered in the presence of LCK knockdown (Fig. 3C, D). We next investigated whether LCK modulated TRPM8 multimerization, which is critical for TRPM8 channel function, as previously reported [6, 30]. The results showed that overexpressed LCK was markedly enhanced, whereas LCK knockdown decreased TRPM8 multimerization in the presence of DSS (Fig. 3E–H). We also detected the effect of LCK on the binding of intermolecular TRPM8. The results showed that LCK overexpression markedly increased the binding of intermolecular TRPM8, while the increase was significantly reduced by co-application of PP2 (Fig. 3I, J). Moreover, LCK knockdown effectively reduced the binding of intermolecular TRPM8 (Fig. 3, K, L). These data together indicated that LCK affects TRPM8 multimerization but not intramolecular N-C binding, thereby regulating its channel functions.

### LCK enhances the phosphotyrosine of TRPM8 at Y1022

**Fig. 4:**
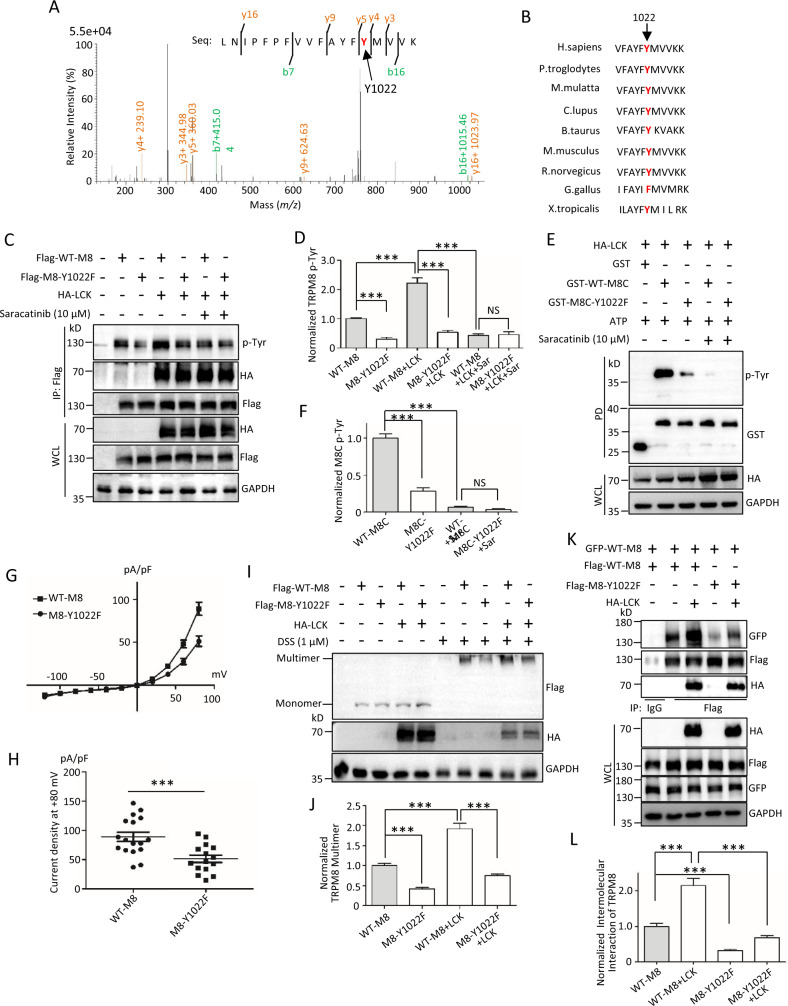
Identification of phosphotyrosine on TRPM8 at position 1022 regulated by LCK. **A** MS imaging of phosphotyrosine site of TRPM8 in combination with the NCBI blast (peptide sequences are indicated). **B** Amino acid sequence alignment showing that tyrosine at position 1022 is highly conserved among multiple species. **C**, **D** Expression constructs for Flag-tagged wild-type TRPM8 (Flag-WT-M8) or mutant Y1022F (Flag-M8-Y1022F) were transfected with or without HA-LCK into HEK293T cells, before harvest for treatment with 10 μM saracatinib for 24 h. The lysates were then used for IP with an anti-Flag antibody and then subjected to WB assay with the indicated antibodies to detect the level of TRPM8 phosphotyrosine. **E**, **F** Kinase assay in vitro. Purified GST alone, GST tagged wild-type or mutant of C-terminus of TRPM8 fusion proteins expressing in E. coli bacteria were mixed with HA-LCK immunoprecipitated with anti-HA antibody from HEK293T cells expressing HA-LCK construct, 1 mM ATP or their combination in kinase assay buffer to determine the level of M8C phosphotyrosine. **G** Relationship of test potential and averaged densities of I_TRPM8_ recorded from HEK293T cells co-transfected Flag-WT-M8 or Flag-M8-Y1022F with pEGFP-N1. **H** Peak current density on +80 mV as in ***G*** (*n* = 15~20 cells per group). **I**, **J** Expression constructs for Flag-WT-M8 or Flag-M8-Y1022F were co-transfected with or without HA-LCK into PANC-1 cells, before harvest for treatment with 1 μM DSS for 30 min for WB to detect the level of TRPM8 multimerization. **K**, **L** Flag-WT-M8 or Flag-M8-Y1022F along with GFP-tagged wild-type TRPM8 (GFP-WT-M8) were co-transfected with or without HA-LCK into AsPC-1 cells to determine the binding of intermolecular TRPM8. ***P < 0.001, NS not significant. Data are presented as mean ± SEM. All studies were repeated at least three times.

We next determined which of the potential phosphotyrosine site(s) in TRPM8 was regulated by LCK. Using the combination of immunoprecipitation and MS analysis, we identified a lysine residue at 423 as a major ubiquitination site of TRPM8 [10]. Within the same screen, the highly conserved tyrosine residue at 1022 across species was also detected as a potential phosphotyrosine site in TRPM8 (Fig. 4A, B). To confirm and characterize the importance of Y1022 for TRPM8 phosphotyrosine, an expression construct for the single-point mutant TRPM8-Y1022F was generated. Compared to wild-type TRPM8 (WT-TRPM8), mutant TRPM8-Y1022F significantly reduced its phosphotyrosine level with or without LCK overexpression (Fig. 4C, D). The significant differences in phosphorylation levels between WT-TRPM8 and mutant TRPM8-Y1022F in the presence of LCK overexpression were abolished by saracatinib (Fig. 4C, D), a potent and selective inhibitor of Src-family tyrosine kinases (SRC, YES and LCK) [31]. An in vitro Kinase assay (Fig. 4E, F) further supported the conclusion that Y1022 in TRPM8 is a potent phosphorylation target for LCK. Interestingly, we found that mutant TRPM8-Y1022F significantly decreased serine phosphorylation (phosphoserine) but did not alter threonine phosphorylation (phosphothreonine) of TRPM8 (Fig. S3A, B). Moreover, LCK significantly enhanced TRPM8 phosphoserine and phosphotyrosine but not phosphothreonine (Fig. S3C, D).

We next recorded the cation currents mediated by mutant TRPM8-Y1022F in HEK293T cells and found that mutant TRPM8-Y1022F decreased I_TRPM8_ densities across at depolarization when compared with WT-TRPM8 (Fig. 4G). At +80 mV, mutant TRPM8-Y1022F markedly decreased I_TRPM8_ densities to ~53% (Fig. 4H). In addition, mutant TRPM8-Y1022F did not affect the expression of total and PM TRPM8 with or without LCK (Fig. S3E–G). WB assays showed that mutant TRPM8-Y1022F significantly reduced TRPM8 multimerization with or without LCK compared to WT-TRPM8 (Fig. 4I, J). The mutant TRPM8-Y1022F significantly impaired the interaction of intermolecular TRPM8 (Fig. 4K, L), further supporting the importance of Y1022 for TRPM8 multimerization. Together, these data suggested that mutant TRPM8-Y1022F modulated channel function likely by modulating TRPM8 multimerization, confirming the effects of LCK on TRPM8 function and multimerization.

### 14-3-3ζ mediates LCK in the regulation of TRPM8 multimerization

**Fig. 5:**
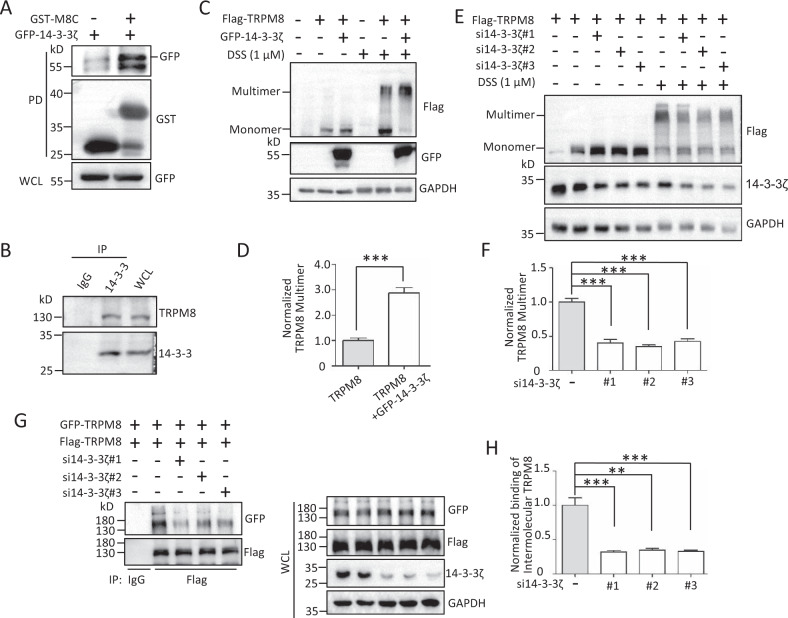
14-3-3ζ interaction with TRPM8 for regulating TRPM8 multimerization. **A** GST pull-down assay. Purified GST alone or GST-M8C fusion proteins expressed in E. coli BL21 bacteria were incubated with the lysates from HEK293T cells expressing GFP-14-3-3ζ constructs and subjected to WB assay. **B** Co-IP assay. The lysates of native PANC-1 cells were added to an anti-14-3-3 antibody for IP and then subjected to WB assay with an anti-TRPM8 antibody. **C**, **D** Expression constructs for Flag-TRPM8 with or without GFP-14-3-3ζ were transfected into PANC-1 cells, before harvest for treatment with 1 μM DSS for 30 min. The lysates were subjected to WB assay with the indicated antibodies to detect the level of TRPM8 multimerization. **E**, **F** Similar experiments in (**C**) and (**D**) but cells co-expressing Flag-TRPM8 along with human 14-3-3ζ-specific siRNAs (si14-3-3ζ#1, #2, or #3). **G**, **H** AsPC-1 cells were co-transfected Flag-TRPM8 and GFP-TRPM8 with or without si14-3-3ζ, and then harvested for IP with an anti-Flag antibody and WB assay with the indicated antibodies to determine the binding of intermolecular TRPM8. **P < 0.01, ***P < 0.001. Data are presented as mean ± SEM. All studies were repeated at least three times.

The 14-3-3 protein is a widely expressed acidic protein that binds with phosphorylated targeted proteins and enhances its multimerization to regulate its activities [32]. 14-3-3ζ, a member of the 14-3-3 protein family, was identified in the same screen at ~30 kD bands as in our previous studies in Fig. 1a [10] (Fig. S1E, F). We first demonstrated the interaction of 14-3-3ζ and the C-terminus of TRPM8 by an in vitro GST pull‐down assay (Fig. 5A) and 14–3–3ζ and TRPM8 in native PANC-1 cells by Co-IP assay (Fig. 5B), suggesting that TRPM8 interacted with 14-3-3ζ in a protein complex. Next, we determined the functional role of 14-3-3ζ in TRPM8 multimerization. The results showed that 14-3-3ζ overexpression or knockdown markedly enhanced or reduced TRPM8 multimerization, respectively, in the presence of DSS (Fig. 5C–F). 14-3-3ζ knockdown effectively inhibited the binding of intermolecular TRPM8 in AsPC-1 cells (Fig. 5G, H), further supporting the role of 14-3-3ζ in TRPM8 multimerization.

**Fig. 6:**
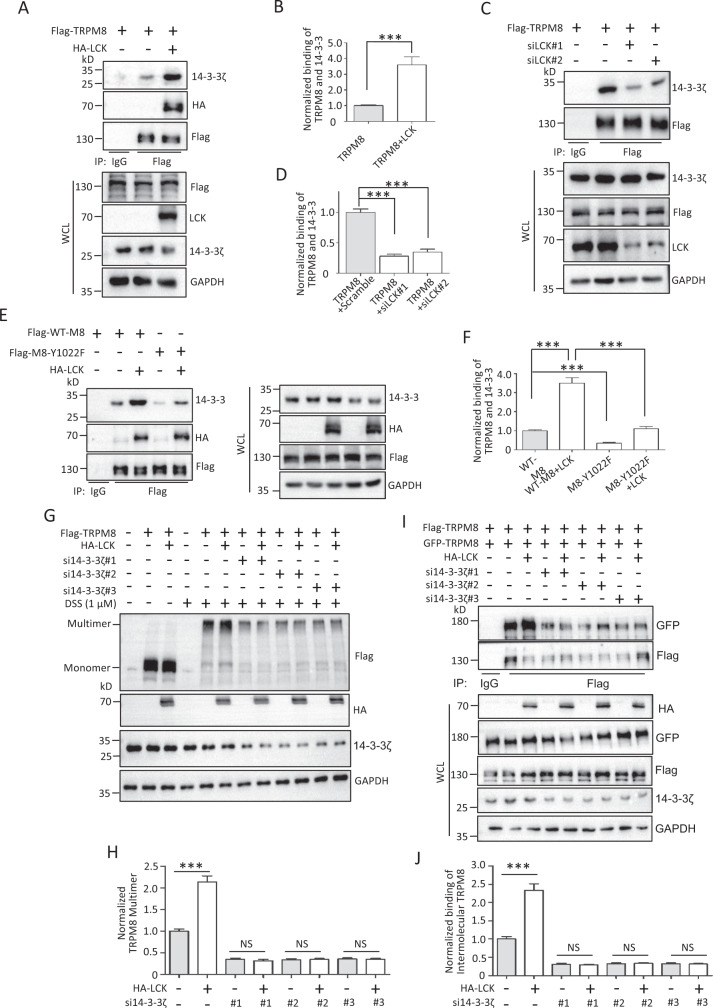
14-3-3ζ involved in the regulation of LCK on the multimerization of TRPM8. **A**, **B** PANC-1 cells were co-transfected Flag-TRPM8 with HA-LCK or control vector and then harvested for IP with an anti-Flag antibody and WB assay with the indicated antibodies to determine the binding of TRPM8 and 14-3-3ζ. **C**, **D** Similar experiments in (**A**) and (**B**) but cells co-expressing Flag-TRPM8 along with siLCK. **E**, **F** Expression constructs for Flag-WT-TRPM8 or Flag-TRPM8-Y1022F were co-transfected with or without HA-LCK into HEK293T cells to determine the binding of TRPM8 and 14-3-3ζ. **G**, **H** PANC-1 cells were co-transfected with Flag-TRPM8, HA-LCK, si14-3-3ζ (#1, #2 or #3) or their combination, before harvest for treatment with 1 μM DSS for 30 min, and subjected to WB assay to detect the level of TRPM8 multimerization. **I**, **J** AsPC-1 cells were co-transfected with Flag-TRPM8 and GFP-TRPM8, HA-LCK, si14-3-3ζ or their combination, and then harvested for IP with an anti-Flag antibody and subjected to determine the binding of intermolecular TRPM8. ***P < 0.001, NS not significant. Data are presented as mean ± SEM. All studies were repeated at least three times.

Based on the above findings, we hypothesized that 14-3-3ζ is involved in the regulation of TRPM8 multimerization by LCK. We first determined whether LCK-mediated TRPM8 phosphotyrosine affected the binding of TRPM8 and 14-3-3ζ. The results showed that LCK overexpression and knockdown significantly increased and reduced the binding of TRPM8 and 14-3-3ζ, respectively (Fig. 6A–D). Meanwhile, mutant TRPM8-Y1022F significantly impaired the binding of 14-3-3ζ and TRPM8 in the presence or absence of LCK (Fig. 6E, F). These data suggested that TRPM8 phosphotyrosine regulated by LCK positively modulated the 14-3-3ζ-TRPM8 interaction. Moreover, 14-3-3ζ knockdown eliminated the function of LCK in increasing TRPM8 multimerization (Fig. 6G, H), which was further validated by the impaired interaction of intermolecular TRPM8 regulated by LCK in the presence of 14-3-3ζ knockdown (Fig. 6I, J). Together, these data revealed that 14-3-3ζ is critical for LCK-mediated regulation of TRPM8 multimerization.

### TRPM8 phosphotyrosine positively modulates LCK activity

**Fig. 7:**
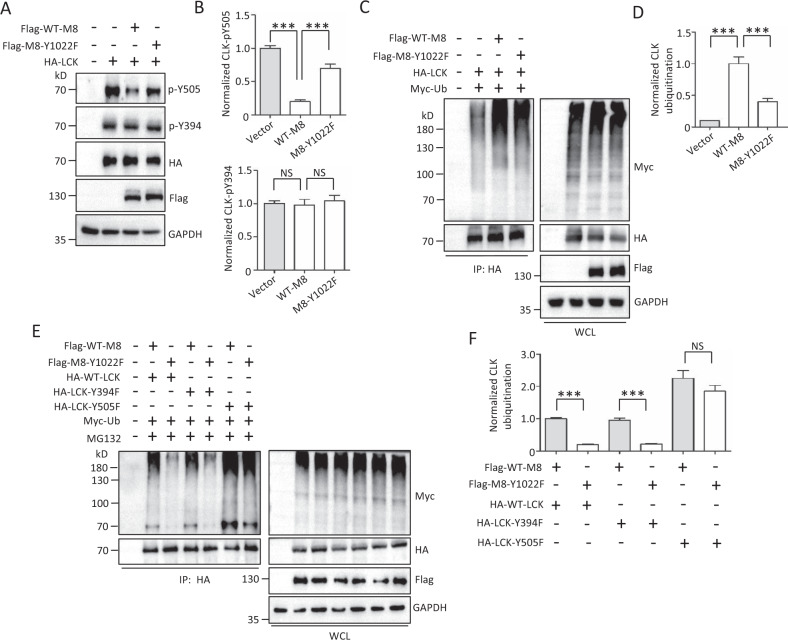
LCK activity regulated by TRPM8 phosphotyrosine. **A**, **B** LCK phosphorylation assay. PANC-1 cells were co-transfected HA-LCK with the control vector, WT-TRPM8 or mutant TRPM8-Y1022 with the indicated antibodies as shown in (**A**), and quantification of the level of LCK phosphorylation shown in (**B)**. **C**–**F** LCK ubiquitination assay. **C**, **D** Expression constructs for HA-LCK and Myc-Ub were co-transfected with control vector, WT-TRPM8 or mutant TRPM8-Y1022 into AsPC-1 cells. The cells were treated with 10 μM MG132 for 6 h before harvest and used for IP with an anti-HA antibody and WB with the indicated antibodies. **E**, **F** Expression constructs for Myc-Ub and WT-TRPM8 or mutant TRPM8-Y1022F were co-transfected with HA-tagged wild-type or mutant LCK into PANC-1 cells. The cells were treated with 10 μM MG132 before harvest and used for IP with an anti-HA antibody and WB with the indicated antibodies. ***P < 0.001, NS not significant. Data are presented as mean ± SEM. All studies were repeated at least three times.

Phosphorylation of Tyr394 or Tyr505 is critical for the regulation of LCK activity [20–22], as validated by our data showing that the Y394F and Y505F mutants, respectively, significantly inhibited and enhanced the function of LCK on TRPM8 phosphotyrosine (Fig. S4A, B). Next, we detected whether TRPM8 phosphotyrosine feedback modulated LCK activity and phosphorylation site-specific antibodies against LCK on Y394 and Y505 were used to detect the levels of LCK Y394 and Y505 phosphorylation, which were specific and effective (Fig. S5A). The results revealed that the level of phosphorylated LCK on Y394 was comparable between cells overexpressing the control vector, WT-TRPM8, and TRPM8-Y1022F (Fig. 7A, B). However, WT-TRPM8 markedly reduced the level of phosphorylated LCK on Y505 compared to the control, while the reduction was countered in the presence of mutant Y1022F-TRPM8 (Fig. 7A, B). Meanwhile, TRPM8 rarely affected LCK Ser/Thr phosphorylation (Fig. S5B, C). These data together suggested that TRPM8 phosphotyrosine positively modulated LCK activity through inhibition of phosphorylated LCK on Y505. Meanwhile, TRPM8 overexpression significantly decreased the level of phosphorylated LCK on Y505 but not Y394, with or without saracatinib. However, saracatinib markedly enhanced the level of phosphorylated LCK on Y505 (Fig. S5D, E), suggesting that saracatinib inhibited LCK activity likely through the activation of Tyr505 phosphorylation.

In addition to phosphorylation, ubiquitination is involved in the regulation of LCK activity [23–25]. Ubiquitination assays showed that WT-TRPM8 overexpression significantly increased LCK ubiquitination compared with the control vector, whereas the increase was partially impaired in the presence of mutant Y1022F-TRPM8 (Fig. 7C, D), suggesting that TRPM8 phosphotyrosine feedback modulated LCK ubiquitination. Moreover, we also detected the effect of TRPM8 phosphotyrosine on the ubiquitination of the LCK mutants Y394F and Y505F. Compared to WT-TRPM8, mutant TRPM8-Y1022F showed similar inhibitory effects on the ubiquitination of WT-LCK and mutant LCK-Y394F (Fig. 7E, F). However, the inhibitory effect of TRPM8-Y1022F on LCK ubiquitination was almost abrogated in the presence of mutant LCK-Y505F (Fig. 7E, F), suggesting that TRPM8 phosphorylation modulated the ubiquitination of the inactive form of LCK (LCK-Y394F).

### Impaired phosphorylation of TRPM8 inhibits pancreatic cancer cell proliferation, migration, and tumorigenesis in vitro and in vivo

**Fig. 8:**
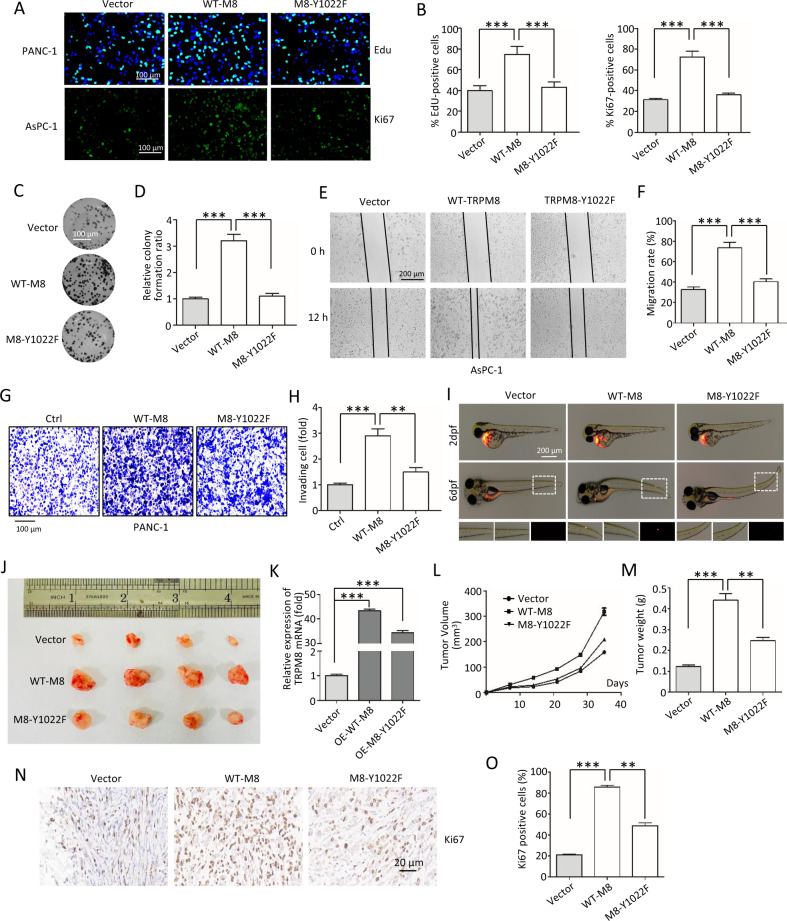
The role of Y1022 on TRPM8 on tumor cell proliferation, migration, and tumorigenesis. **A**–**D** Cell proliferation assays in vitro. **A**, **B** The RFP labeled cell lines of PANC-1 or AsPC-1 cells stably expressing control vector, WT-TRPM8, or mutant TRPM8-Y1022F were constructed and used for EdU incorporation assays (Upper panel) and Ki67 immunofluorescence (Lower panel). Scale bars, 100 µm. **C**, **D** Colony formation assays were performed in RFP labeled PANC-1 stably maintained cells. Scale bars, 100 µm. **E**–**I** Cell migration assays. Scale bars, 200 µm. **E**, **F** Wound-healing assay was performed in RFP labeled AsPC-1 stably maintained cells. **G**, **H** Transwell assay was performed in RFP labeled PANC-1 stably maintained cells. Scale bars, 100 µm. **I**–**L** Animal xenotransplantation engraftment experiments. **I** Representative confocal microscopy images of 6 days xenotransplantation of zebrafish injecting with RFP labeled PANC-1 stably maintained cells. Scale bars, 200 µm. **J** Imaging of tumors excised from the mice subcutaneously injecting RFP labeled PANC-1 stably maintained cells by growth for 5 weeks. **K** Quantification of the expression of TRPM8 mRNA in (**J**). **L** Weights of the excised tumors in each group in (**J**). **M** Growth curves showing the changes in the tumor volume in mice in different groups every 5 days from the injection. **N** Representative H&E staining images and immunohistochemical images of Ki67 in excised tumors tissues. Scale bars, 20 µm. **O** Quantification of Ki67 expression in (**N**). **P < 0.01, ***P < 0.001. Data are presented as mean ± SEM. All studies were repeated at least three times.

We next examined the effect of Y1022 in TRPM8 on pancreatic cancer cell proliferation and migration using of RFP-labeled PANC-1 or AsPC-1 cells stably expressing control vector, WT-TRPM8, or mutant TRPM8-Y1022F. EdU incorporation assay, immunofluorescence, and colony formation together revealed that WT-TRPM8 significantly increased tumor cell proliferation when compared to control vector cells. However, mutant TRPM8-Y1022F impaired the function of TRPM8 on increasing cell proliferation (Fig. 8A–D). These data suggested that TRPM8 phosphorylation at Y1022 is critical for pancreatic cancer cell proliferation.

In addition, wound-healing and transwell assays revealed that WT-TRPM8 showed a significantly higher migration capacity than control vector cells, whereas mutant TRPM8-Y1022F impaired the function of TRPM8 on cell migration (Fig. 8E–H). We also employed a novel metastatic zebrafish xenotransplantation model to detect the effect of mutant TRPM8-Y1022F on tumor cell migration. After 6 days of xenotransplantation, compared to the control group, a large number of PANC-1 cells stably expressing WT-TRPM8 migrated to distant parts of the zebrafish body to form micrometastases, while PANC-1 cells stably expressing mutant TRPM8-Y1022F did not migrate far from the primary site (Fig. 8I). Together, these data suggest that TRPM8 phosphorylation at Y1022 is critical for pancreatic cancer cell migration.

To further assess the importance of TRPM8 phosphorylation in Y1022 tumorigenesis in vivo, BALB/c nude mice bearing subcutaneous pancreatic xenograft tumors derived from PANC-1 cells stably expressing control vector, WT-TRPM8, and mutant TRPM8-Y1022F was used. After 35 days of growth, the tumors were carefully removed (Fig. 8J). TRPM8 mRNA expression detected by real-time qRT-PCR was up-regulated in the tumor xenografts stably expressing WT-TRPM8 or TRPM8-Y1022F (Fig. 8K). Moreover, compared to the control xenograft tumors, a significant increase in tumor volumes and weights was observed in WT-TRPM8 xenograft tumors and Ki67 expression was markedly increased in WT-TRPM8 xenograft tumor tissues by histopathologic analyses (Fig. 8L–O). However, mutant TRPM8-Y1022F diminished the increase in WT-TRPM8 tumor volumes and weights as well as Ki67 expression. Together, these data suggest that TRPM8 phosphorylation at Y1022 contributes to tumorigenesis in vivo.

## Discussion

TRPM8, which functions as a Ca^2+^-permeable channel, requires the assembly of functional homologous tetramers [6–8] and plays a vital role in environmental cold sensing, menthol-induced analgesia of acute and inflammatory pain, and migraines [33]. Elevated expression of TRPM8 has been found in human pancreatic cancer and several other diseases in clinical patients [34]. However, how the TRPM8 channel in PM exerts its oncogenic effects is not well understood. Moreover, phosphotyrosine is involved in the regulation of TRPM8 function but the exact site(s) is unkonown [16]. In the present study, we identified LCK and 14-3-3ζ as new TRPM8 binding partners and a novel post-translational modification of TRPM8 at Y1022. Moreover, we provided a novel model in which LCK-mediated TRPM8 phosphorylation at Y1022 is critical for I_TRPM8_ density by enhancing 14-3-3ζ-TRPM8 binding to regulate of TRPM8 multimerization. Knockdown of 14-3-3ζ markedly impaired the regulation of TRPM8 multimerization by LCK. In addition, phosphorylation and ubiquitination mediated LCK activity was coordinately regulated by TRPM8 phosphotyrosine at Y1022 in a feedback loop. Importantly, we provided multiple lines of evidence supporting the importance of TRPM8 phosphotyrosine at Y1022 on pancreatic cancer progression in vitro and in vivo.

Earlier studies showed that a member of the Src family kinases Src, but not Abl or Btk, phosphorylates TRPM8 and modulates the cold‐induced activation of the TRPM8 channel by using a combination of a constitutively active isoform of Src, Src inhibitor, and Src siRNA, without detecting the interaction of TRPM8 and Src [16]. In this study, we employed GST pull-down in combination with MS assays and found that BLK, LCK, and LYN were potential interacting partners of TRPM8, which was further strengthened by Co-IP assays. However, ~60 kD of Src was failed to be detected in a similar size band of BLK, LCK, and LYN. We speculated that Src regulates TRPM8 function in an indirect manner. LCK has emerged as one of the key molecules that functions in lymphocytes and stimulates several ion channels, especially the Kv1.3 potassium channel [35–39]. LCK coupled with hDlg indirectly regulated Kv1.3 channel activities [40]. Apart from Kv1.3, it is not clear whether LCK stimulates other channels in a direct or indirect manner. Our data revealed that LCK directly interacted with TRPM8 by protein-protein interaction assay in vitro and positively modulated TRPM8 phosphotyrosine and I_TRPM8_ densities, expanding the mechanism of LCK function on ion channels. Notably, BLK or LYN interacted with TRPM8, but did not affect TRPM8 phosphotyrosine or I_TRPM8_ densities. Thus, the physiological role of the TRPM8 interaction with BLK or LYN should be assessed.

TRPM7, belonging to the TRPM family with TRPM8, binds to 14-3-3, which requires autophosphorylation of TRPM7 at S1403 [29]. However, 14-3-3 is involved in regulating TRPM7 cellular localization [29], exhibiting a clear difference in the regulation of TRPM8 multimerization. In addition, our data revealed the importance of 14-3-3ζ for LCK and impaired phosphorylation of TRPM8 (TRPM8-Y1022F) regulating channel multimerization, supporting the idea that TRPM8 phosphotyrosine modulates 14-3-3ζ-mediated channel multimerization. Previous studies have shown that binding of 14-3-3 to proteins usually occurs after phosphorylation of Ser/Thr within two conserved consensus motifs (RSXpS/TXP or RXXXpS/TXP), leading to various functional consequences for regulating its activation or deactivation [27, 28]. Apart from phosphotyrosine, LCK, as a tyrosine kinase, also increased the level of TRPM8 phosphoserine. TRPM8-Y1022F markedly inhibited the level of TRPM8 phosphoserine, further supporting that TRPM8 phosphotyrosine affected its own phosphoserine level. Thus, we speculated that TRPM8 phosphoserine might be involved in the process of 14-3-3ζ-mediated channel multimerization regulated by LCK, although the exact phosphoserine site(s) involved require further study. Together, after phosphorylating TRPM8 at Y1022, LCK coordinately enhanced the TRPM8 phosphoserine for recruiting 14-3-3ζ and providing cross-bridging of 14-3-3ζ and TRPM8 in a complex, leading to TRPM8 multimerization for elevated I_TRPM8_ densities on the PM.

LCK, as a Src family tyrosine kinase, was originally identified as playing an important role in T-cell functions [17, 18]. Currently, LCK has been shown to function as an oncogene in leukaemia and various solid cancers, including breast cancer, colon cancer, and lung carcinoma [41]. Indeed, several LCK inhibitors have been approved to treat leukemia and various solid cancers, including pancreatic cancer [42–45]. LCK activity is mainly regulated via phosphorylation/dephosphorylation of crucial tyrosine residues Y394 and Y505 [20–22]. Our data revealed that TRPM8 phosphotyrosine feedback altered the level of phosphorylated LCK on Y505 but not Y394, which is responsible for the elevated activity of LCK. Therefore, TRPM8 phosphotyrosine suppressed the phosphorylation of LCK on Y505 in the inactive state, thereby enhancing LCK activity. Several studies revealed that ubiquitination is also involved in the regulation of LCK activity in a diverse manner [23–25]. Heat shock protein 90 (Hsp90) prevents the active form Y505F of mutant LCK from being targeted for degradation by ubiquitination [23]. Apart from HSP90-mediated LCK ubiquitination without altering LCK expression, other regulators, such as Cbl and SOCS-6, modulate the degradation of LCK [23–25]. TRPM8 phosphorylation positively modulated LCK ubiquitination without affecting LCK expression, especially the ubiquitination of the inactive form Y394F of mutant LCK. Thus, we elucidated the mechanism by which TRPM8 mediates ubiquitination at inactive LCK to regulate its kinase activity, which is different from previous studies [23]. Nevertheless, how TRPM8 coordinates the crosstalk of phosphorylation and ubiquitination across LCK in different active states to modulate its activity needs further investigation.

There are limitations to the present study. Although TRPM8-Y1022F markedly affected its own phosphoserine level, we could not distinguish TRPM8 phosphotyrosine directly or indirectly regulating 14-3-3ζ-mediated channel multimerization regulated by LCK, which requires further study on the involved exact phosphoserine site(s) of TRPM8. In addition, future studies are needed to determine the degree of TRPM8 multimerization mediated by LCK is positively correlated with the severity of pancreatic cancer in clinical patients.

## Conclusions

**Fig. 9:**
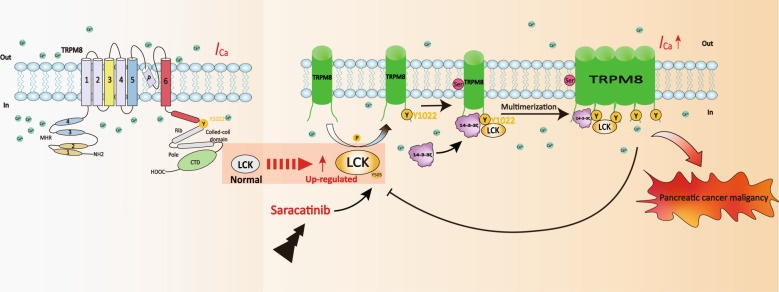
Schematic diagram. Schematic diagram of the biological role of the LCK-14-3-3ζ-TRPM8 axis in the regulation of TRPM8 function and LCK activity.

In summary, we investigated the molecular determinants and functional consequences of TRPM8 phosphotyrosine (Fig. 9). The four main findings are as follows: (1) the Src family tyrosine kinase LCK is a novel TRPM8‐interacting protein that phosphorylates TRPM8 at Y1022 and elevates I_TRPM8_ densities. (2) LCK and TRPM8 phosphotyrosine at Y1022 modulated I_TRPM8_ densities by modulating TRPM8 multimerization, which is involved in 14-3-3ζ-TRPM8 binding and the regulation of 14-3-3ζ on TRPM8 multimerization. (3) Y1022 in the C‐terminal of TRPM8 is a critical phosphorylation residue involved in the regulation of the proliferation and migration of pancreatic cancer cells. (4) TRPM8 phosphotyrosine feedback modulates LCK activity by regulating the crosstalk of phosphorylation and ubiquitination. These data establish a link between LCK, 14-3-3ζ and TRPM8 and provide mechanistic insights into the LCK-14-3-3ζ-TRPM8 axis for a full understanding of TRPM8 multimerization mediated channel function and LCK activity. Targeting the inhibition of the LCK-14-3-3ζ-TRPM8 axis to impair oncogene function of both TRPM8 and LCK may enhance tumor sensitivity to therapeutics, allowing for potential pharmacological targeting for anticancer therapy.

## Materials and methods

### Antibodies and reagents

Rabbit anti-LCK (#12477, PTGCN, China), anti-GFP (#50430, PTGCN), anti-14-3-3 (#14503, PTGCN), anti-phosphotyrosine (p-Tyr) (P4110, Sigma), anti-phosphothreonine (#9391, Cell Signaling Technology), anti-phosphoserine (Abcam, ab9332), anti-phospho-Lck-Y505 (pY505) (#MAB7500, R& D), anti-TRPM8 (#ACC-049, Alomone, Israel), and mouse anti-phospho-Lck-Y394 (pY394) (#2751, Cell Signaling Technology) antibodies were used at a dilution factor of 1:1000. A mouse anti-GFP antibody (#66002, PTGCN), anti-HA (#M180, MBL, Japan), anti-Flag (#M185, MBL), anti‐Myc (#M192, MBL), and anti-GAPDH (#60004, PTGCN) were used at a dilution factor of 1:3000. A goat anti-rabbit or anti-mouse HRP-conjugated secondary antibody obtained from Millipore was used at a dilution factor of 1:20,000. The compounds for 4-amino-5-(4-chlorophenyl)-7-(dimethylethyl) pyrazolo[3,4-d] pyrimidine (PP2), sodium orthovanadate (Na_3_VO_4_), disuccinimidyl suberate (DSS), MG132, and saracatinib were obtained from Selleck. All reagents for cell culture were obtained from Invitrogen.

### Cell culture and transfection

The human cervical cancer cell line HeLa, human embryonic kidney 293T (HEK293T), and human pancreatic cancer cell lines PANC-1 and AsPC-1 were obtained and cultured as previously described [46]. All cell lines were maintained in Dulbecco’s modified essential medium (DMEM) containing 10% fetal bovine serum (FBS), L-glutamine (2 mM), penicillin G (100 units/ml), and streptomycin (10 mg/ml) at 37 °C with 5% CO_2_. Cells with 60~70% confluent were transfected with the indicated expression construct or siRNA using Lipofectamine™ 2000 Transfection Reagent (Invitrogen) according to the manufacturer’s instructions. After 48 h of transfection, the cells were harvested for follow-up corresponding experiments.

### Constructs and siRNA

The expression constructs for full-length rat Trpm8 (NM_134371) in pcDNA3 (pcDNA3-TRPM8), pcDNA3.1-N-Flag (Flag-TRPM8), and pEGFP-N1 (GFP-TRPM8) were previously described [10, 47]. The truncated expression constructs for the GST fused C-terminus of TRPM8 (GST-M8C) in pGEX-4T-1, GFP fused C-terminus of TRPM8 (GFP-M8C) in pEGFP-C1, and Flag-tagged cytosolic domains of TRPM8 (1-691 for M8-N, 756–759 for M8-LII, 815–829 for M8-LI, and 980–1104 for M8-C) subcloned into the pCMV10-3×Flag vector were previously described [47]. The expression constructs for mutant TRPM8 with mutation Y1022F was introduced using a PCR-based mutagenesis method [10, 48, 49]. The human BLK, LCK, and LYN cDNAs were kindly provided by Prof. Jiahuai Han (Xiamen University, China) and subcloned into pcDNA3.1-HA to express HA-fused BLK, LCK, and LYN in mammalian cells. LCK cDNA was also subcloned into pET28α(+) to express His-fused LCK (His-LCK) in E. coli BL21. Human 14-3-3ζ cDNA from HEK293T cells was subcloned into pEGFP-C1 to express GFP-fused 14-3-3ζ in mammalian cells. All expression constructs were verified by direct DNA sequencing analysis. The siRNA targeting human LCK (siLCK#1: 5′-UCAAGAACCUGAGCCGCAATT-3′ and siLCK#2: 5′-GGCAGCCCAAAUUGCAGAATT-3′), siRNA targeting human 14-3-3ζ (si14-3-3#1: 5′-GCCUGCAUGAAGUCUGUAATT-3′, si14-3-3#2: 5′-CGUCUCAAGUAUUGAACAATT-3′ and si14-3-3#3: 5′-CACGCUAAUAAUGCAAUUATT-3′), and scrambled control siRNA (5’-UUCUCCGAACGUGUCACGUTT-3′) were designed and synthesized by GenePharma (Suzhou, Jiangsu, China). The siRNA knockdown efficiency was verified using western blotting analysis with an anti-LCK and anti-14-3-3 antibody. TRPM8 mRNA was detected by real-time qRT-PCR using the following primers: Forward: 5′-TCTGCCGACCTTCAGGAGGT-3′, Reverse: 5′-ATGGAGTTCCACATCCAAGTCC-3′.

### Lentiviral production and creation of stable cell lines

The lentiviral production and creation of stable cell lines were performed as described previously [46]. The DNA fragment of TRPM8 was subcloned into the lentiviral plasmid pCDH-CMV-MCS-EF1-turboRFP-T2A-Puro. HEK293T cells in 10-cm dishes were transfected with 5 μg of lentiviral constructs together with viral packaging plasmids, 3 μg of psPAX2 and 3 μg of pMD2.G (all related viral plasmids were kindly provided by Prof. Xiaorong Zhang). After 48 h of transfection, the viral supernatant was harvested, filtered through a 0.22 μm filter, and then added to PANC-1 or AsPC-1 cells in 6-cm dishes with 10 μg/μl polybrene (Solarbio, H8761). At 48 h of viral infection, the cells were selected and cultured by replacing a new culture medium containing puromycin (Solarbio, IP1280) every 3–4 days for several weeks. Clones of puromycin-resistant cells stably expressing TRPM8 were isolated, characterized, and expanded in complete culture medium supplemented with 2 μg/ml puromycin.

### Western blot and immunoprecipitation

Western blotting (WB) and immunoprecipitation were performed following the procedure described previously [49]. Briefly, cells were lysed with lysis buffer (50 mM Tris-HCl, pH 7.5, 150 mM NaCl, 0.5% NP-40, and 1 mM EDTA supplemented with 1× protease inhibitor complete mini EDTA-free cocktail from Roche). The supernatants of cell lysates were boiled for 5 min in 1× SDS loading buffer (6×, 0.3 M Tris–HCl, 6% SDS, 60% glycerol, 120 mM dithiothreitol (DDT), and a proprietary pink tracking dye), subjected to 8–15% sodium dodecyl sulfate-polyacrylamide gel electrophoresis (SDS-PAGE) and transferred to polyvinylidene difluoride (PVDF) membrane. After blocking with 5% nonfat dry milk in TBST (20 mM Tris-HCl, 150 mM NaCl, 0.05% Tween-20), the membrane was then incubated with the indicated primary antibodies, secondary antibodies, and SuperSignal West Pico Plus (Invitrogen, 34,580) according to the manufacturer’s instructions. Finally, the protein signal on the membrane was recorded using a ChemiDoc XRS system (Bio-Rad Laboratories, Richmond, CA) and analyzed using the Image Lab software (Bio-Rad Laboratories).

For immunoprecipitation, 2 μg of the indicated antibody together with 500 μl of cell lysates (500 μg) were mixed at 4 °C for 3 h, followed by incubation with 30 μl of Protein-A/G beads (Santa Cruz Biotechnology) for 2 h. After three washes three times with lysis buffer supplemented with 0.1% Tween, the resulting immunocomplexes were subjected to WB assay. Studies were repeated at least three times.

For the ubiquitination assay, cells treated with 10 μM proteasome inhibitor MG132 for 6 h were harvested and lysed with the denaturation buffer (6 M guanidine-HCl, 0.1 M Na_2_HPO_4_/NaH_2_PO_4_, 10 mM imidazole) as described previously [47]. The supernatant of lysates was mixed with the indicated antibody and then with Protein‐A/G beads for 3 h with rotation at RT, followed by washes and WB assay.

### GST pull-down assay and protein-protein interaction assay in vitro

The GST pull-down assay was performed as described previously [47]. Recombinant proteins for GST-M8C or GST alone expressed in E. coli BL21 cells were purified using glutathione beads according to the manufacturer’s protocol (Thermo). Purified GST-M8C or GST alone was incubated with protein lysates extracted from HEK293T cells overexpressing HA-BLK, HA-LCK or HA-LYN. After four washes with lysis buffer, the complex of protein-bound GST-agarose beads was washed four times with sonication buffer (0.5% Nonidet P-40, 50 mM Tris/HCl, 150 mM NaCl, 1 mM EDTA supplemented with 1× protease inhibitor cOmplete Mini EDTA-free mixture from Roche) and subjected to WB assay.

For the protein-protein interaction assay in vitro, the expression of the His-LCK fusion protein in E. coli BL21 cells was induced with 1 mM isopropyl 1-thio-β-D-galactopyranoside (IPTG) for 8 h at 20 °C. The cells were then harvested, resuspended in sonication buffer, and sonicated on ice. Following centrifugation, the supernatants were incubated with the Ni-NTA agarose (Beyotime, Shanghai, China) with rotation for 3 h at 4 °C. The immobilized His-LCK was then washed with sonication buffer and 2 and 5 mM imidazole. Elution with 50 mM imidazole and 150 μg of bound His-LCK proteins were incubated with the above purified the complex of GST or GST-M8C protein-bound GST-agarose beads for 1 h at room temperature (RT). After washing with sonication buffer four times, the complex of protein-bound GST-agarose beads was subjected to WB assay.

### Kinase assay in vitro

The LCK kinase assay in vitro experiments was performed using a modified protocol as previously described [50, 51]. LCK proteins extracted from HEK293T cells overexpressing HA-LCK were immunoprecipitated with anti-HA antibody. The immune-complex was extensively washed with lysis buffer twice, washed with kinase assay buffer (20 mM Tris-HCl pH 7.5, 10 mM MgCl_2_, 10 mM MnCl_2_), and incubated together with 100 ng of bacterially purified GST-M8C in kinase assay buffer supplemented with 1 mM ATP. After incubation for 30 min at 37 °C for 30 min, the reaction mixtures were terminated and boiled for 5 min in 1× SDS sample buffer, followed by WB assay to detect the phosphotyrosine of the C-termini of TRPM8.

### Electrophysiological experiments

For electrophysiological experiments, whole-cell cation currents mediated by TRPM8 (I_TRPM8_) were recorded by whole-cell patch-clamp technologies with an Axon MultiClamp 700B amplifier using the Digidata1550A digitizer (Axon Instruments, Sunnyvale, CA) as described previously [10]. Briefly, the indicated expression constructs pEGFP-N1 and Flag-TRPM8 were transfected into HEK293T cells. After 48 h of transfection, we selected the cells expressing an approximately equal amount of GFP to record I_TRPM8_ at RT in an extracellular solution containing (mM) 150 NaCl, 6 CsCl, 1 MgCl_2_, 1.5 CaCl_2_, 10 glucose, 10 mM HEPES, pH 7.4 with NaOH. The peptides were filled with pipette solution ((mM): 150 NaCl, 3 MgCl_2_, 5 EGTA, 10 HEPES, pH 7.2 with NaOH) to form a tip resistance of 2~4 MΩ. Series resistance was compensated by 75–85% to reduce voltage errors. The holding potential was −60 mV, and details of each pulse protocol are given schematically in the related figures. The densities of the whole-cell TRPM8 current (I_TRPM8_) were normalized to the cell capacitance (pA/pF). The data were analyzed using a combination of Clampfit version 11.0 (Molecular Devices), Microsoft Excel, and GraphPad Prism 5 (GraphPad Software Inc., San Diego, CA, USA).

### Cell surface biotinylation assay

Isolation of PM proteins by cell surface biotinylation assay was described previously [10, 48, 49]. Briefly, cells were harvested and incubated with sulfo-NHS-SS-Biotin to label the PM proteins in ice-cold PBS for 30 min, followed by incubation with 100 mM glycine to quench the biotinylation reaction. After three washes with PBS, the cells were harvested in the above lysis buffer. The biotinylated proteins were precipitated with NeutrAvidin-agarose resin beads (Pierce) overnight at 4 °C. The protein-bead complex was washed with lysis buffer and then resuspended in 1× SDS loading buffer for WB assays.

### Immunocytochemistry and confocal microscopy

Immunocytochemistry was performed as described previously [47, 52]. Transfected HEK293T, HeLa or ASPC-1 cells on glass coverslips were washed three times with ice-cold PBS, fixed for 10 min with 4% paraformaldehyde (w/v) in PBS, and permeabilized for 15 min by incubation with 0.2% Triton X-100 at RT. After blocking with 1× PBS containing 0.1% Triton X-100 (v/v) and 10% goat serum (v/v) for 2 h, the samples were incubated with the indicated primary antibodies (e.g., anti-Ki67 antibody (#27309, PTGCN)) overnight at 4 °C and fluorescence-labeled secondary antibodies in PBS supplemented with 2% FBS and 1% BSA for 2 h at RT. DAPI (1 μg/ml, Solarbio, C0065) was used for nuclear staining. Finally, the samples were washed three times with ice-cold PBS and observed with a confocal laser-scanning microscope (Leica SP8, Wetzlar, Germany). At least three fields of view were analyzed. Data analysis was performed using the Leica LAS AF Lite software.

### 5-Ethynyl-20-deoxyuridine (EdU) incorporation assay

The EdU incorporation assay was performed as described previously [52]. EdU-labeled transfected PANC-1 cells were examined using the BeyoClick™ EdU Cell Proliferation Kit with Alexa Fluor 488 (Beyotime, C0071S) and then imaged under an Olympus FSX100 microscope.

### In vitro colony formation assay

A colony formation assay was performed as described previously [47, 52]. Approximately equal amounts of PANC-1 cells transfected with vector, wild-type or mutant TRPM8 were seeded in 12-well plates and allowed to grow for 7~10 days. The medium was replaced every 3 days. Cells were washed twice with PBS, fixed with 4% paraformaldehyde, and stained with 0.5% crystal violet staining solution (Sigma-Aldrich, USA). Colonies with more than 50 cells in triplicate wells were counted.

### In vitro cell migration assay

The effects of TRPM8 on the migration ability of cells were evaluated using wound-healing and Transwell assays. For the wound-healing assay, stably maintained AsPC-1 cells with 80% confluent in 12-well plates were cultured for 24 h after the formation of a monolayer. The monolayer was scratched with the tip of a 10 μL pipette and washed with PBS to remove the cell fragments, followed by the addition of the conditioned medium. The wound healed for 12 h and was imaged at the same wound location using an Olympus FSX100 microscope.

For the Transwell assay, ~5 × 10^4^ of transfected cells were digested and placed into the upper chamber precoated with an 8 μm pore Transwell insert (Fisher Scientific, 0877121) with the lower chamber containing medium (containing 10% FBS). After incubation for 24 h at 37 °C and 5% CO_2_. The upper surface of the membrane was then gently scraped using a cotton swab to remove the non migrated cells and washed twice with PBS. The wells were then fixed in 4% paraformaldehyde for 30 min, permeabilized with 0.2% Triton for 10 min, and stained with 0.5% crystal violet staining solution. Following two washes with PBS, the migrated cells were observed and photographed under an Olympus FSX100 microscope. The number of migrated cells was determined by averaging five random fields per well.

### Animal xenotransplantation engraftment experiments

Animal experiments in our study have been reviewed and approved for the use of laboratory animals by the Hubei University of Technology Animal Care and Use Committee. The zebrafish were maintained according to standard protocols (http://ZFIN.org), and embryos were grown at 28.5 °C in egg water (60 μg/ml Instant Ocean sea salts). For zebrafish engraftment xenotransplantation, 2 days post-fertilization (dpf), embryos were utilized and obtained from adult AB zebrafish (Danio rerio). Approximately 300 of RFP-labeled stably maintained PANC-1 cells were inoculated into the blood circulation of 2 dpf zebrafish embryos as previously described [53]. Prior to microinjection, the survival rate of cells was above 90% by analyzing some cells using trypan blue staining and counting. Embryos were incubated at 34 °C for 4 days and imaged under anesthesia in egg water containing 200 μg/ml tricaine (Sigma Aldrich) using fluorescence microscopy (Leica M205FA, Germany).

For xenograft engraftment in mice, BALB/c nude mice at 4–6 weeks of age (18–22 g) were utilized and purchased from Vital River Laboratory Animal Technology (Beijing, China). Three million stably maintained PANC-1 cells in 100 μl of phosphate-buffered saline (PBS) were subcutaneously implanted into the left and right axillae of female BALB/c nude mice per group and grown for 4–6 weeks as previously described [46, 52]. The tumor volume (*V*) was monitored and measured every 5 days with the following formula: *V* = [(tumor length × width × 2)/2], and the weight was calculated when the mice were sacrificed.

### Statistical analysis

Data are presented as the mean ± SEM, and all data reported are based on at least three independent experiments. Student’s unpaired or paired two-tailed *t* tests (GraphPad) were performed to determine statistical significance as appropriate. For comparisons of more than two groups, one-way analysis of variance was employed for normal distributions, and the Kruskal–Wallis test was employed for nonnormal or small samples. *P* values < 0.05 were considered statistically significant. *Represents *P* < 0.05, **represents *P* < 0.01 and ***represents *P* < 0.001, NS stands for “not significantly different”.

### Reporting summary

Further information on research design is available in the Nature Research Reporting Summary linked to this article.

## Supplementary information

Supplementary Figures 1-5

Full length uncropped original western

Reporting Summary

## Data Availability

Please contact the corresponding author (Jingfeng_HUT@163.com or cefan@hbut.edu.cn) for data requests.

## References

[1] Vincent A, Herman J, Schulick R, Hruban RH, Goggins M (2011). Pancreatic cancer. Lancet..

[2] Kobi M, Veillette G, Narurkar R, Sadowsky D, Paroder V, Shilagani C (2020). Imaging and management of pancreatic cancer. Semin Ultrasound CT MR.

[3] McKemy DD, Neuhausser WM, Julius D (2002). Identification of a cold receptor reveals a general role for TRP channels in thermosensation. Nature..

[4] Yee NS, Brown RD, Lee MS, Zhou W, Jensen C, Gerke H (2012). TRPM8 ion channel is aberrantly expressed and required for preventing replicative senescence in pancreatic adenocarcinoma: potential role of TRPM8 as a biomarker and target. Cancer Biol Ther.

[5] Yee NS (2016). TRPM8 ion channels as potential cancer biomarker and target in pancreatic cancer. Adv Protein Chem Struct Biol.

[6] Erler I, Al-Ansary DM, Wissenbach U, Wagner TF, Flockerzi V, Niemeyer BA (2006). Trafficking and assembly of the cold-sensitive TRPM8 channel. J Biol Chem.

[7] Tsuruda PR, Julius D, Minor DJ (2006). Coiled coils direct assembly of a cold-activated TRP channel. Neuron..

[8] Phelps CB, Gaudet R (2007). The role of the N terminus and transmembrane domain of TRPM8 in channel localization and tetramerization. J Biol Chem.

[9] Asuthkar S, Velpula KK, Elustondo PA, Demirkhanyan L, Zakharian E (2015). TRPM8 channel as a novel molecular target in androgen-regulated prostate cancer cells. Oncotarget..

[10] Huang Y, Li S, Jia Z, Li S, He W, Zhou C (2021). TRIM4 interacts with TRPM8 and regulates its channel function through K423-mediated ubiquitination. J Cell Physiol.

[11] Gkika D, Lemonnier L, Shapovalov G, Gordienko D, Poux C, Bernardini M (2015). TRP channel-associated factors are a novel protein family that regulates TRPM8 trafficking and activity. J Cell Biol.

[12] Rohacs T, Lopes CM, Michailidis I, Logothetis DE (2005). PI(4,5)P2 regulates the activation and desensitization of TRPM8 channels through the TRP domain. Nat Neurosci.

[13] Zheng W, Cai R, Hofmann L, Nesin V, Hu Q, Long W (2018). Direct binding between Pre-S1 and TRP-like domains in TRPP channels mediates gating and functional regulation by PIP2. Cell Rep.

[14] Abe J, Hosokawa H, Sawada Y, Matsumura K, Kobayashi S (2006). Ca2+-dependent PKC activation mediates menthol-induced desensitization of transient receptor potential M8. Neurosci Lett.

[15] Morgan K, Sadofsky LR, Crow C, Morice AH (2014). Human TRPM8 and TRPA1 pain channels, including a gene variant with increased sensitivity to agonists (TRPA1 R797T), exhibit differential regulation by SRC-tyrosine kinase inhibitor. Biosci Rep.

[16] Manolache A, Selescu T, Maier GL, Mentel M, Ionescu AE, Neacsu C (2020). Regulation of TRPM8 channel activity by Src-mediated tyrosine phosphorylation. J Cell Physiol.

[17] Adler HT, Reynolds PJ, Kelley CM, Sefton BM (1988). Transcriptional activation of lck by retrovirus promoter insertion between two lymphoid-specific promoters. J Virol.

[18] Voronova AF, Sefton BM (1986). Expression of a new tyrosine protein kinase is stimulated by retrovirus promoter insertion. Nature..

[19] Kumar SP, Kashyap A, Silakari O (2018). Exploration of the therapeutic aspects of Lck: a kinase target in inflammatory mediated pathological conditions. Biomed Pharmacother.

[20] Yamaguchi H, Hendrickson WA (1996). Structural basis for activation of human lymphocyte kinase Lck upon tyrosine phosphorylation. Nature..

[21] Peri KG, Gervais FG, Weil R, Davidson D, Gish GD, Veillette A (1993). Interactions of the SH2 domain of lymphocyte-specific tyrosine protein kinase p56lck with phosphotyrosine-containing proteins. Oncogene..

[22] Eck MJ, Atwell SK, Shoelson SE, Harrison SC (1994). Structure of the regulatory domains of the Src-family tyrosine kinase Lck. Nature..

[23] Giannini A, Bijlmakers MJ (2004). Regulation of the Src family kinase Lck by Hsp90 and ubiquitination. Mol Cell Biol.

[24] Rao N, Miyake S, Reddi AL, Douillard P, Ghosh AK, Dodge IL (2002). Negative regulation of Lck by Cbl ubiquitin ligase. Proc Natl Acad Sci USA.

[25] Choi YB, Son M, Park M, Shin J, Yun Y (2010). SOCS-6 negatively regulates T cell activation through targeting p56lck to proteasomal degradation. J Biol Chem.

[26] Fu H, Subramanian RR, Masters SC (2000). 14-3-3 proteins: structure, function, and regulation. Annu Rev Pharm Toxicol.

[27] Gardino AK, Smerdon SJ, Yaffe MB (2006). Structural determinants of 14-3-3 binding specificities and regulation of subcellular localization of 14-3-3-ligand complexes: a comparison of the X-ray crystal structures of all human 14-3-3 isoforms. Semin Cancer Biol.

[28] Liu J, Cao S, Ding G, Wang B, Li Y, Zhao Y (2021). The role of 14-3-3 proteins in cell signalling pathways and virus infection. J Cell Mol Med.

[29] Cai N, Lou L, Al-Saadi N, Tetteh S, Runnels LW (2018). The kinase activity of the channel-kinase protein TRPM7 regulates stability and localization of the TRPM7 channel in polarized epithelial cells. J Biol Chem.

[30] Stewart AP, Egressy K, Lim A, Edwardson JM (2010). AFM imaging reveals the tetrameric structure of the TRPM8 channel. Biochem Biophys Res Commun.

[31] Hennequin LF, Allen J, Breed J, Curwen J, Fennell M, Green TP (2006). N-(5-chloro-1,3-benzodioxol-4-yl)-7-[2-(4-methylpiperazin-1-yl)ethoxy]-5- (tetrahydro-2H-pyran-4-yloxy)quinazolin-4-amine, a novel, highly selective, orally available, dual-specific c-Src/Abl kinase inhibitor. J Med Chem.

[32] Clatot J, Hoshi M, Wan X, Liu H, Jain A, Shinlapawittayatorn K (2017). Voltage-gated sodium channels assemble and gate as dimers. Nat Commun.

[33] Liu B, Fan L, Balakrishna S, Sui A, Morris JB, Jordt SE (2013). TRPM8 is the principal mediator of menthol-induced analgesia of acute and inflammatory pain. Pain..

[34] Liu Z, Wu H, Wei Z, Wang X, Shen P, Wang S (2016). TRPM8: a potential target for cancer treatment. J Cancer Res Clin Oncol.

[35] Lepple-Wienhues A, Szabo I, Laun T, Kaba NK, Gulbins E, Lang F (1998). The tyrosine kinase p56lck mediates activation of swelling-induced chloride channels in lymphocytes. J Cell Biol.

[36] Desai R, Peretz A, Idelson H, Lazarovici P, Attali B (2000). Ca2+-activated K+ channels in human leukemic Jurkat T cells. Molecular cloning, biochemical and functional characterization. J Biol Chem.

[37] Lepple-Wienhues A, Wieland U, Laun T, Heil L, Stern M, Lang F (2001). A src-like kinase activates outwardly rectifying chloride channels in CFTR-defective lymphocytes. FASEB J.

[38] Kuras Z, Kucher V, Gordon SM, Neumeier L, Chimote AA, Filipovich AH (2012). Modulation of Kv1.3 channels by protein kinase A I in T lymphocytes is mediated by the disc large 1-tyrosine kinase Lck complex. Am J Physiol Cell Physiol..

[39] Szigligeti P, Neumeier L, Duke E, Chougnet C, Takimoto K, Lee SM (2006). Signalling during hypoxia in human T lymphocytes-critical role of the src protein tyrosine kinase p56Lck in the O2 sensitivity of Kv1.3 channels. J Physiol.

[40] Hanada T, Lin L, Chandy KG, Oh SS, Chishti AH (1997). Human homologue of the Drosophila discs large tumor suppressor binds to p56lck tyrosine kinase and Shaker type Kv1.3 potassium channel in T lymphocytes. J Biol Chem.

[41] Bommhardt U, Schraven B, Simeoni L (2019). Beyond TCR signaling: emerging functions of Lck in cancer and immunotherapy. Int J Mol Sci.

[42] Chen R, Chen B (2015). The role of dasatinib in the management of chronic myeloid leukemia. Drug Des Devel Ther.

[43] Lindauer M, Hochhaus A (2014). Dasatinib. Recent Results Cancer Res.

[44] Creeden JF, Alganem K, Imami AS, Brunicardi FC, Liu SH, Shukla R (2020). Kinome array profiling of patient-derived pancreatic ductal adenocarcinoma identifies differentially active protein tyrosine kinases. Int J Mol Sci.

[45] Marech I, Patruno R, Zizzo N, Gadaleta C, Introna M, Zito AF (2014). Masitinib (AB1010), from canine tumor model to human clinical development: where we are?. Crit Rev Oncol Hematol.

[46] Zhou C, Liang Y, Zhou L, Yan Y, Liu N, Zhang R (2021). TSPAN1 promotes autophagy flux and mediates cooperation between WNT-CTNNB1 signaling and autophagy via the MIR454-FAM83A-TSPAN1 axis in pancreatic cancer. Autophagy.

[47] Huang Y, Li S, Jia Z, Zhao W, Zhou C, Zhang R (2020). Transient receptor potential melastatin 8 (TRPM8) channel regulates proliferation and migration of breast cancer cells by activating the AMPK-ULK1 pathway to enhance basal autophagy. Front Oncol.

[48] Huang Y, Yang J, Xie W, Li Q, Zeng Z, Sui H (2017). A novel KCND3 mutation associated with early-onset lone atrial fibrillation. Oncotarget..

[49] Huang Y, Wang Z, Liu Y, Xiong H, Zhao Y, Wu L (2016). alphaB-Crystallin Interacts with Nav1.5 and Regulates Ubiquitination and Internalization of Cell Surface Nav1.5. J Biol Chem.

[50] Nika K, Soldani C, Salek M, Paster W, Gray A, Etzensperger R (2010). Constitutively active Lck kinase in T cells drives antigen receptor signal transduction. Immunity..

[51] Moogk D, Zhong S, Yu Z, Liadi I, Rittase W, Fang V (2016). Constitutive Lck activity drives sensitivity differences between CD8+ memory T cell subsets. J Immunol.

[52] Zhou C, Yi C, Yi Y, Qin W, Yan Y, Dong X (2020). LncRNA PVT1 promotes gemcitabine resistance of pancreatic cancer via activating Wnt/beta-catenin and autophagy pathway through modulating the miR-619-5p/Pygo2 and miR-619-5p/ATG14 axes. Mol Cancer.

[53] Tulotta C, He S, Chen L, Groenewoud A, van der Ent W, Meijer AH (2016). Imaging of human cancer cell proliferation, invasion, and micrometastasis in a zebrafish xenogeneic engraftment model. Methods Mol Biol.

