# Supplementary material for: The LCK-14-3-3ζ-TRPM8 axis regulates TRPM8 function/assembly and promotes pancreatic cancer malignancy
Source: Cell Death Dis. 2022 Jun 4;13(6):524. doi: 10.1038/s41419-022-04977-5 (PMC9167300; doi:10.1038/s41419-022-04977-5)
Supplement: Supplementary file 1 — Supplementary Figures 1-5 [file 41419_2022_4977_MOESM1_ESM.docx]

**Supplemental Figure**

**
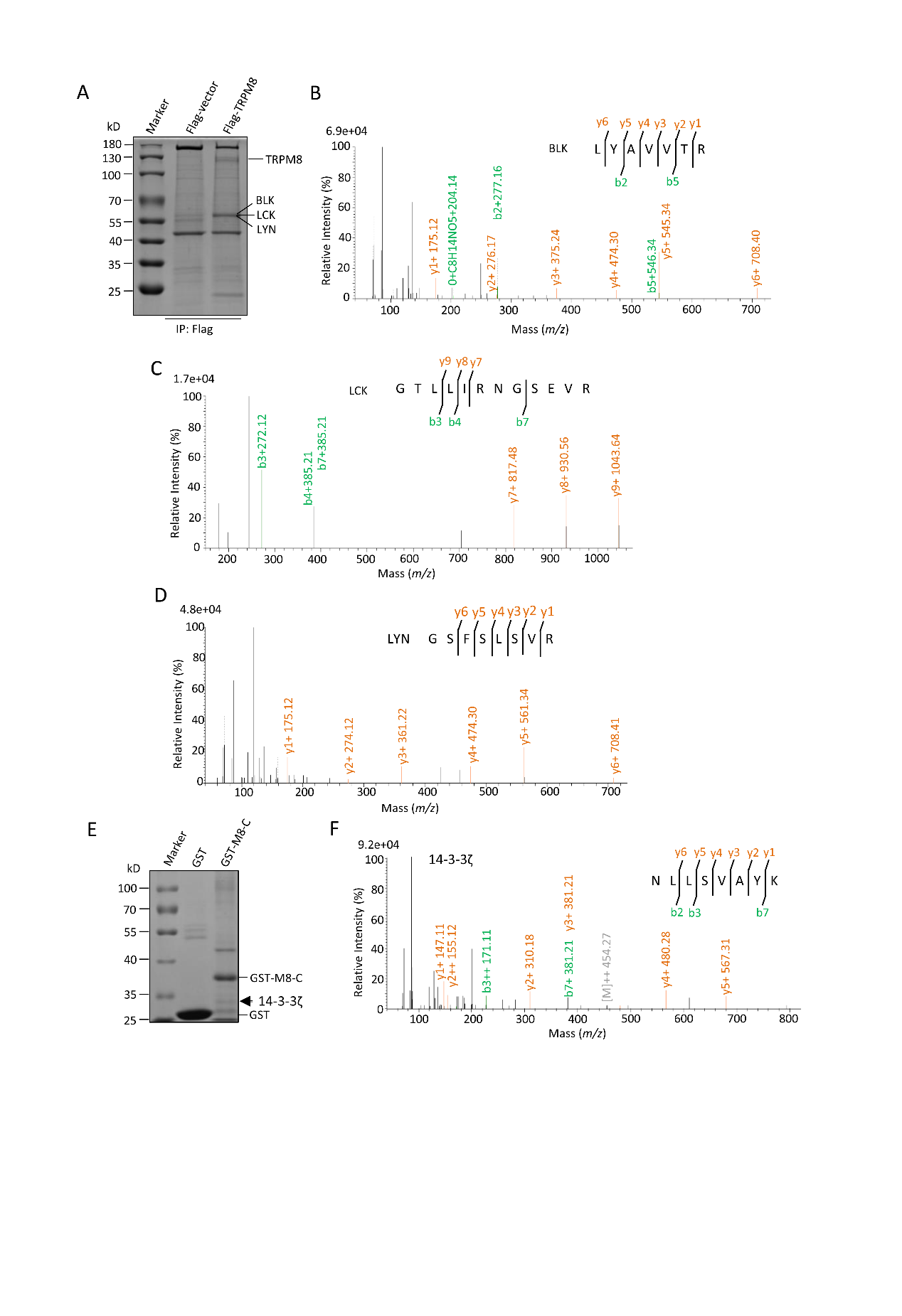
Supplemental Figure 1. Identification of novel TRPM8-interacting partners by Co-IP assay.**

(**A**) Co-IP assay coupled with Coomassie staining. The lysates from MCF7 cells transfected with control vector or Flag-TRPM8 construct were precipitated with an anti-Flag antibody and subjected to Coomassie staining. (**B-D**) The relevant ~ 60 kD band (shown with an arrow) as in ***A*** was selected for mass spectrometric (MS) assay in combination with the NCBI Blast. Peptide sequences of MS assay are indicated in ***B*** (BLK), ***C*** (LCK), and ***D*** (LYN). (**E**) GST pull-down coupled with Coomassie staining. Purified GST alone or GST-M8C proteins expressing in *E.coli* BL21 bacteria were incubated with the lysates of MCF7 cells and subjected to Coomassie staining. The relevant band of ~ 30 kD protein (shown with an arrow) was selected for MS analysis. (**F**) MS imaging of 14-3-3ζ as a novel partner of TRPM8 in combination with the NCBI blast (peptide sequences are indicated).

**
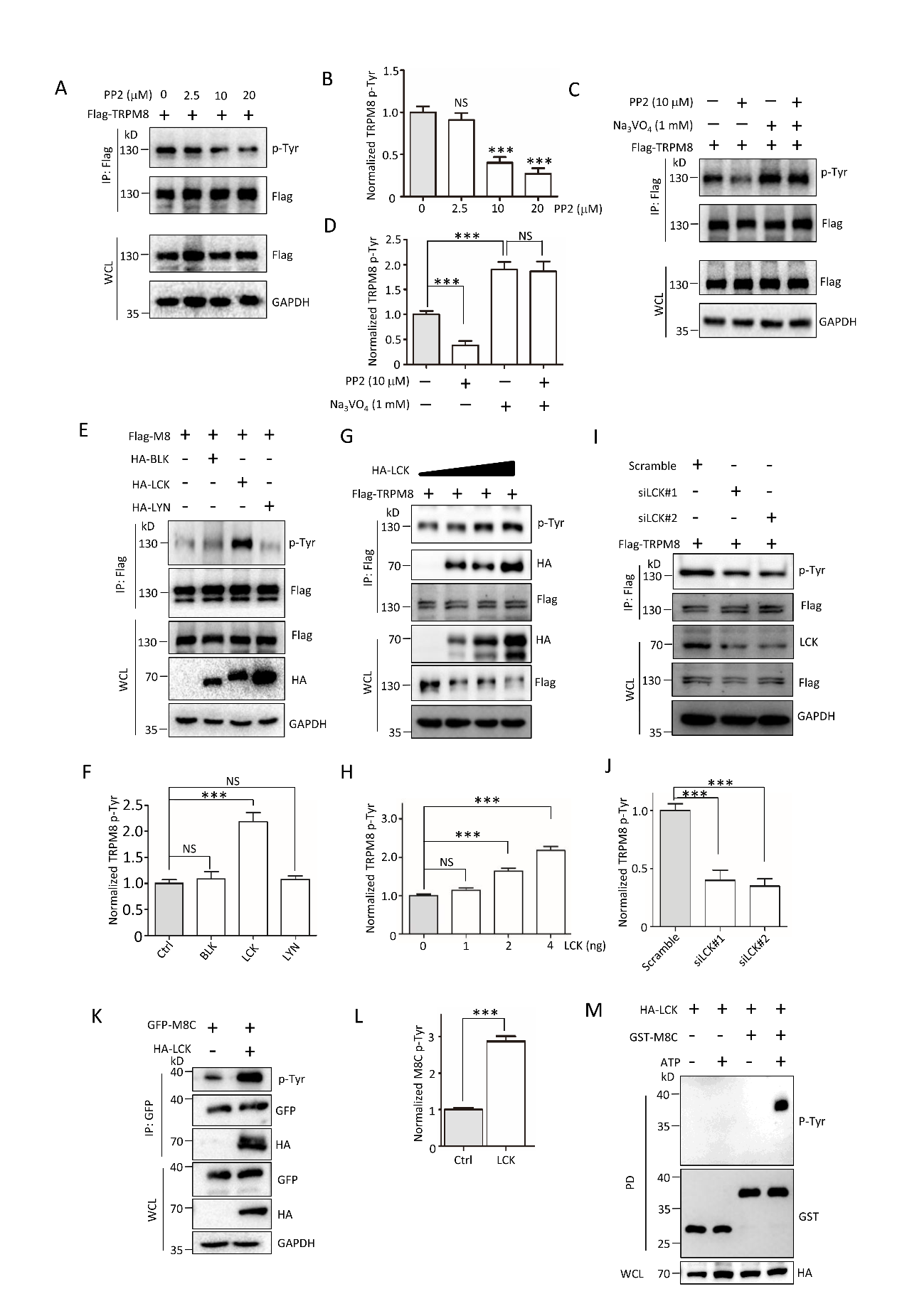
**

**Supplemental Figure 2. Effect of the compounds for PP2 and sodium orthovanadate (Na_3_VO_4_), and LCK on TRPM8 phosphotyrosine.**

(**A**-**B**) HeLa cells expressing Flag-TRPM8 were incubated with DMSO dissolving different concentrations of PP2 (in μM, 0, 2.5, 10, 20) for 24 h before harvest, lysed and immunoprecipitated with an anti-Flag antibody. The samples were then analyzed by immunoblotting with the anti-Flag and p-Tyr antibodies to detect the level of TRPM8 phosphotyrosine. (**C**-**D**) Similar experiments in ***A*** and ***B*** but treatment with 10 μM PP2, 1 mM Na_3_VO_4_, or their combination. Na_3_VO_4_, sodium orthovanadate. PP2, 4-amino-5-(4-chlorophenyl)-7-(dimethylethyl) pyrazolo[3,4-d] pyrimidine, a Src family kinases inhibitor. (**E**-**F**) Expression constructs for Flag-TRPM8 were co-transfected into PANC-1 cells with HA-BLK, HA-LCK, or HA-LYN, respectively. The cells were then harvested for IP with an anti-Flag antibody and WB assay with the anti-Flag and p-Tyr antibodies to detect the level of TRPM8 phosphotyrosine. (**G**-**H**) Similar experiments in ***E*** and ***F*** but cells expressing with various amounts of HA-LCK. (**I**-**J**) Similar experiments in ***E*** and ***F*** but cells expressing with human LCK-specific siRNAs (siLCK#1 or #2) or negative scramble siRNAs. (**K**-**L**) HEK293T cells were co-transfected with GFP-M8C with HA-LCK or control vector, then harvested for IP with an anti-GFP antibodies, and WB assay with the anti-GFP and p-Tyr antibodies to detect the level of M8C phosphotyrosine. (**M**) Kinase assay *in vitro*. Purified GST alone or GST-M8C fusion proteins expressing in *E.coli* bacteria and HA-LCK immunoprecipitated with anti-HA antibody from HEK293T cells expressing HA-LCK constructs were mixed with or without 1 mM ATP in kinase assay buffer (20 mM Tris-HCl pH 7.5, 10 mM MgCl_2_, 10 mM MnCl_2_), and the reaction mixtures were then terminated, followed by WB assay with the indicated antibodies. ***, P < 0.001, NS, not significant. Data are presented as mean ± SEM. All studies were repeated at least three times.

**
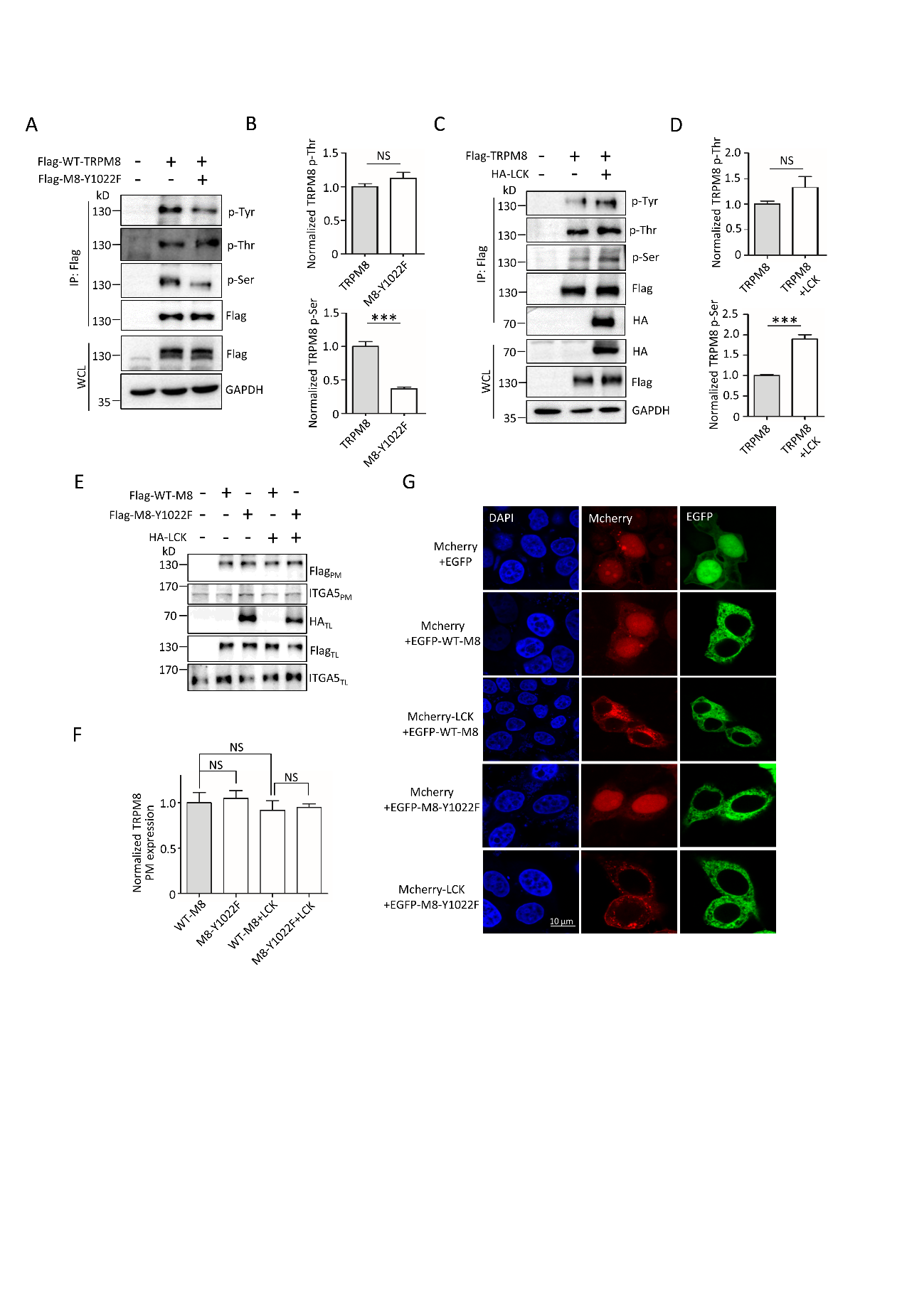
**

**Supplemental Figure 3. The effect of LCK or TRPM8-Y1022F on Ser/Thr phosphorylation of TRPM8 and LCK on mutant TRPM8-Y1022F expression on the PM.**

(**A**-**B**) HEK293T cells were transfected with Flag-WT-TRPM8 or Flag-M8-Y1022F, and harvested for IP with an anti-Flag antibody and WB assay with the indicated antibodies. (**C**-**D**) Flag-TRPM8 were co-transfected with or without HA-LCK into HEK293 cells. The cells were then harvested for IP with an anti-Flag antibody and WB assay with the indicated antibodies. (**E**) WB imaging of TRPM8 in the PM and total lysates from PANC-1 cells co-transfected Flag-WT-TRPM8 or Flag-TRPM8-Y1022F, in the presence or absence of HA-LCK. (**F**) Quantification of PM and total protein expression levels of TRPM8 in (***E***). (**G**) Representative confocal imaging of PANC-1 cells co-expressing mcherry-LCK with EGFP-WT-TRPM8 or EGFP-TRPM8-Y1022F. DAPI (1 μg/ml) was used for nuclei staining. ***, P < 0.001, NS, not significant. Data are presented as mean ± SEM. All studies were repeated at least three

times.


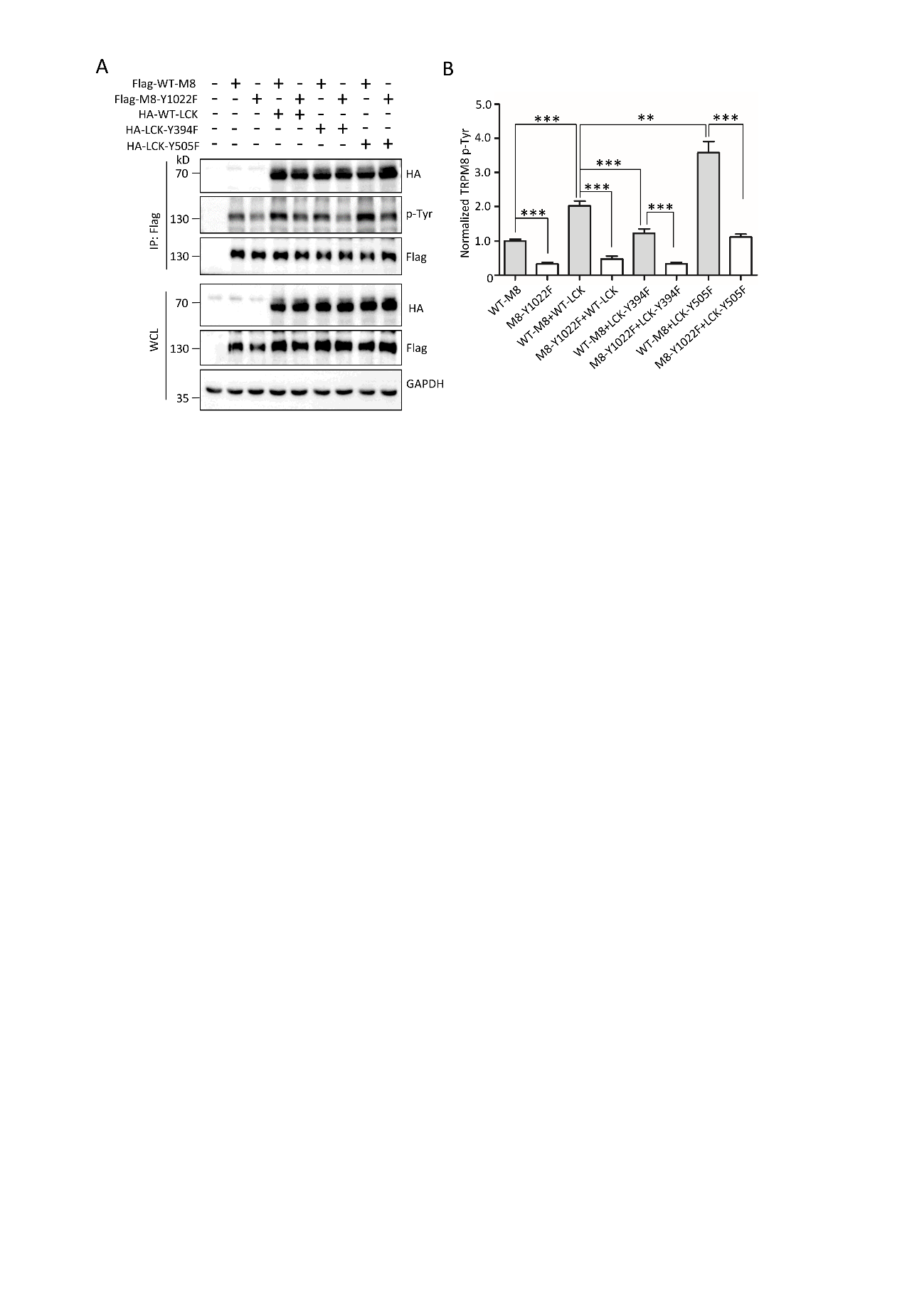


**Supplemental Figure 4. The effect of LCK mutants on TRPM8 phosphotyrosine.**

(**A**-**B**) Expression constructs for Flag-WT-M8 or Flag-M8-Y1022F were co-transfected with control vector, HA-tagged wild type, or mutant LCK into HEK293T cells. The cells were harvested for IP with an anti-Flag antibody and WB with the indicated antibodies to detect the level of TRPM8 phosphotyrosine. **, P < 0.01, ***, P < 0.001, NS, not significant. Data are presented as mean ± SEM. All studies were repeated at least three times.

**
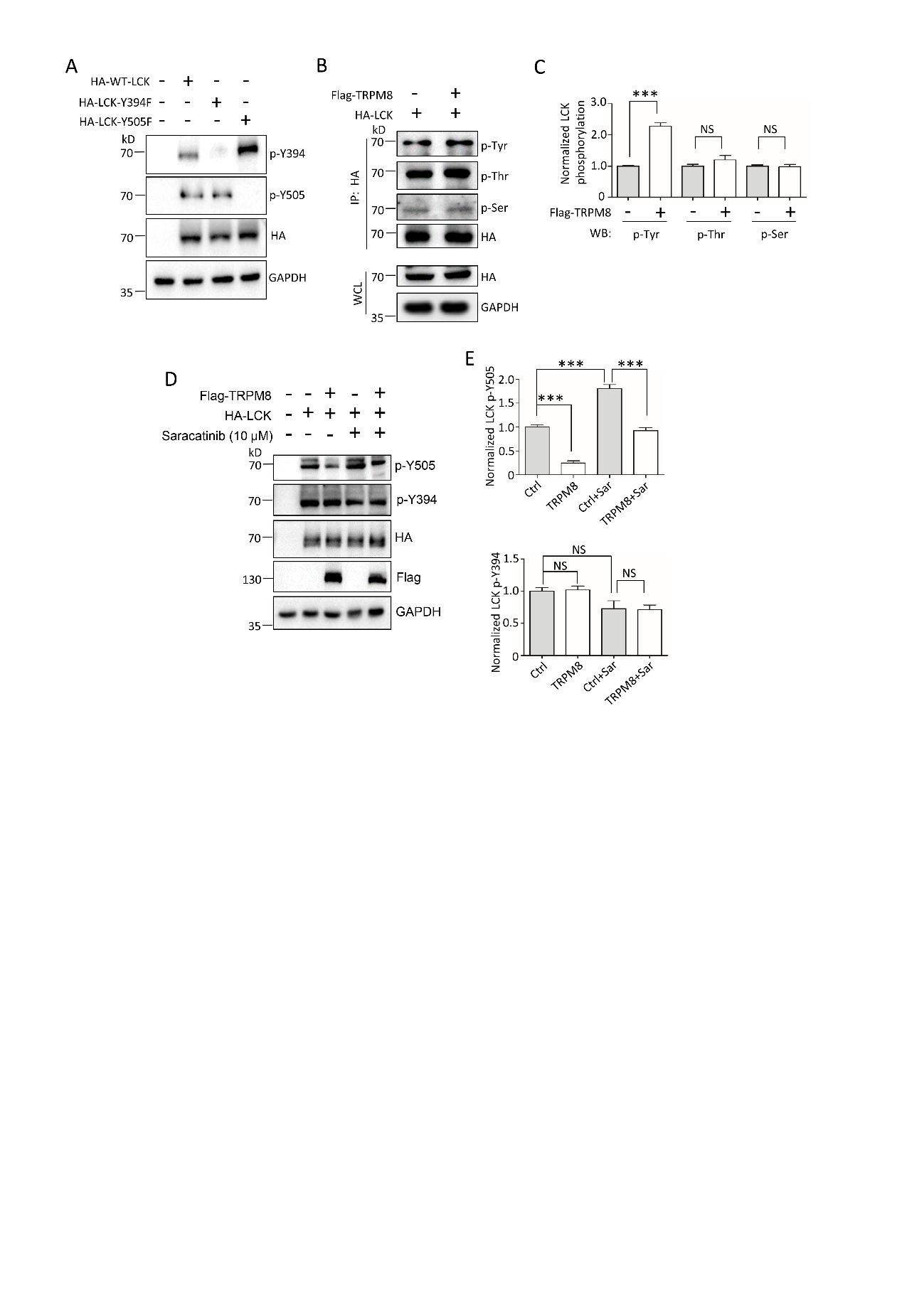
**

**Supplemental Figure 5. The effect of TRPM8 on LCK phosphorylation.**

(**A**) WB imaging of HEK293T cells individually transfected with control vector, HA-tagged wild type, or mutant LCK with the indicated antibodies. (**B**-**C**) PANC-1 cells were co-transfected HA-LCK with or without Flag-TRPM8, and then harvested for IP with an anti-HA antibody and WB assay with the indicated antibodies. (**D**-**E**) PANC-1 cells were co-transfected HA-LCK with or without Flag-TRPM8, before harvest for treatment with 10 μM saracatinib for 24 h. The lysates were subjected to WB assay with the indicated antibodies. ***, P < 0.001, NS, not significant. Data are presented as mean ± SEM. All studies were repeated at least three times.
