# Supplementary figures and images for: The LCK-14-3-3ζ-TRPM8 axis regulates TRPM8 function/assembly and promotes pancreatic cancer malignancy
Source: Cell Death Dis. 2022 Jun 4;13(6):524. doi: 10.1038/s41419-022-04977-5 (PMC9167300; doi:10.1038/s41419-022-04977-5)

Fig. 1

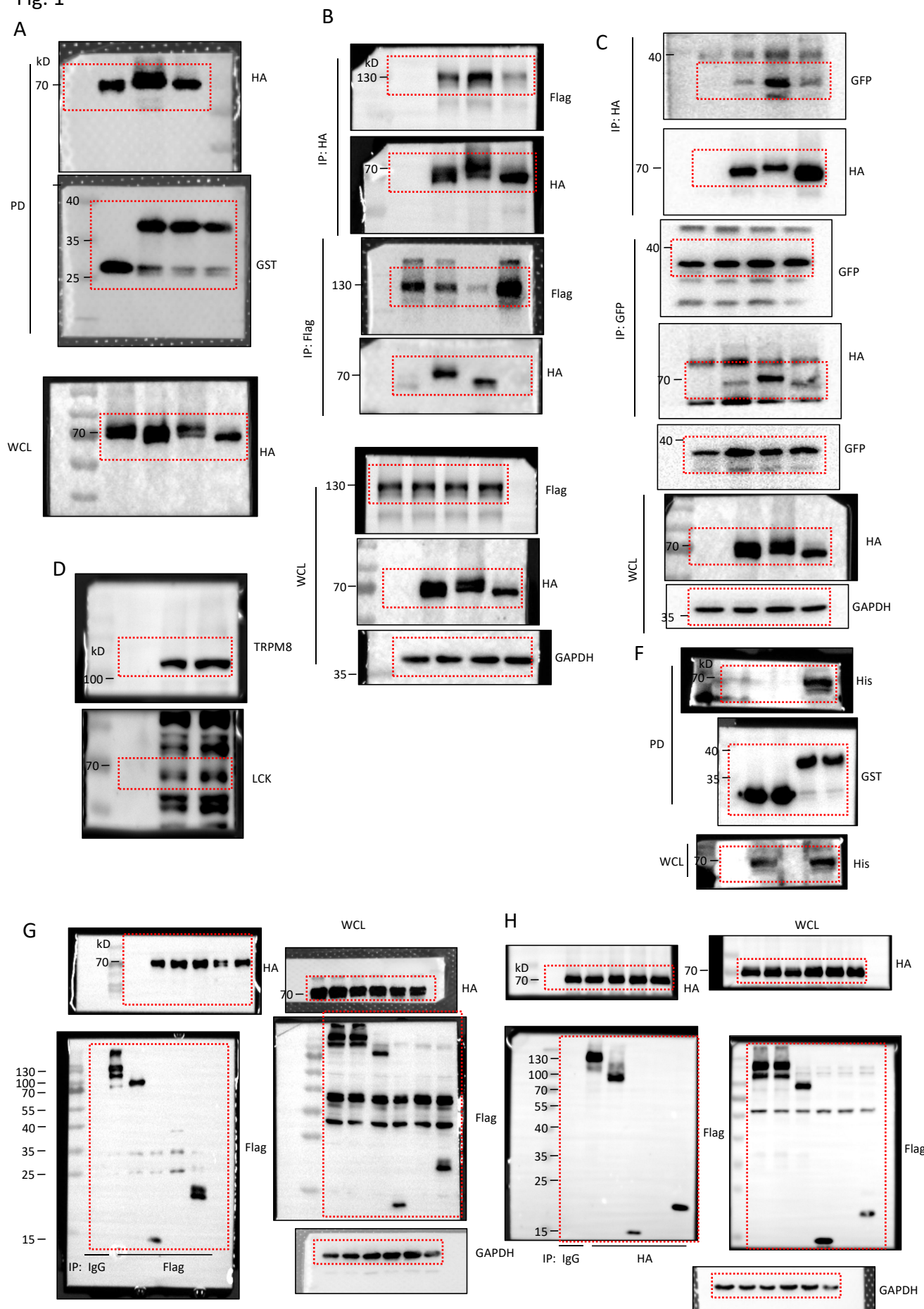

Fig. 2

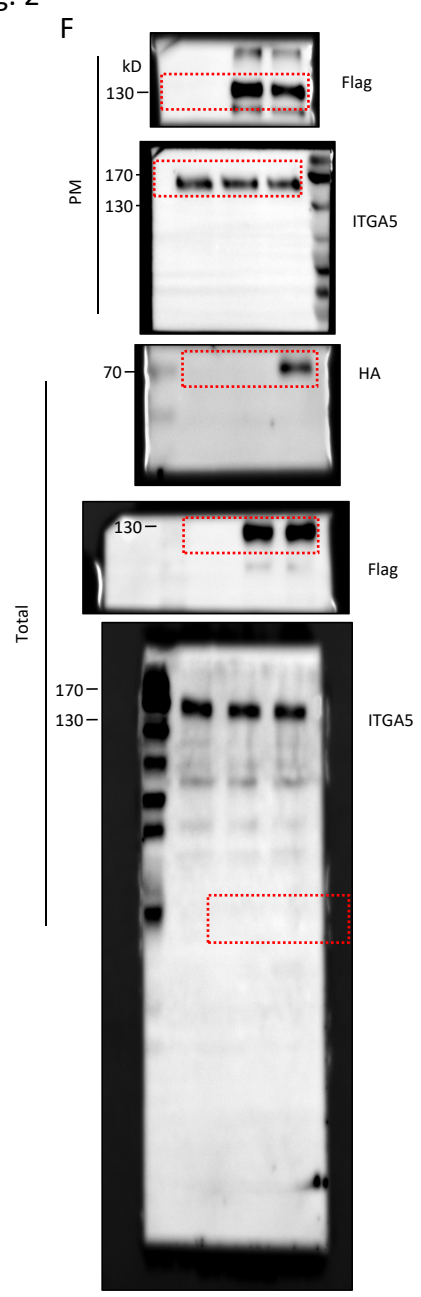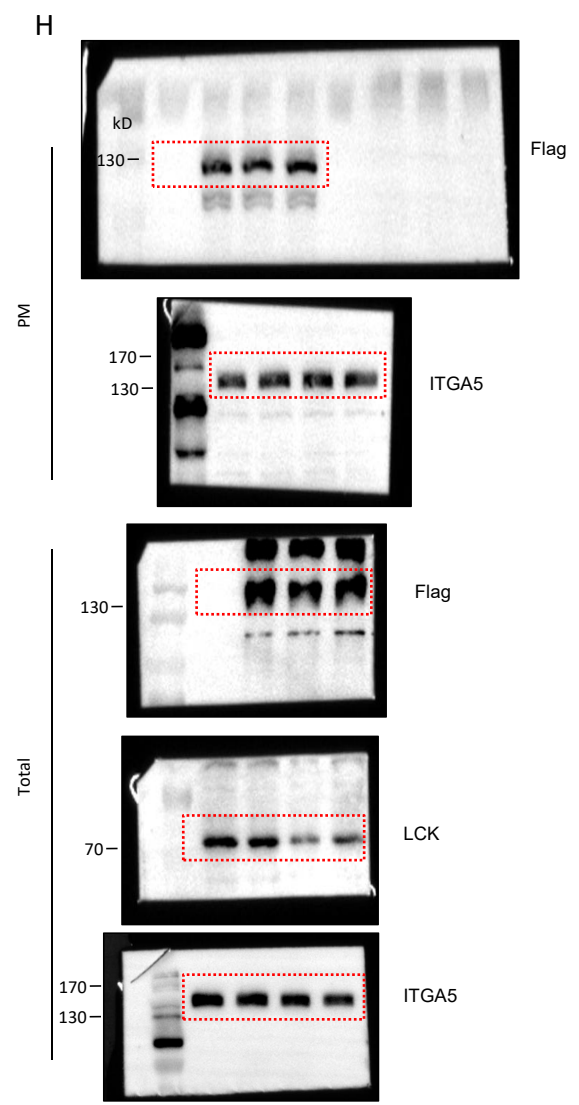

Fig. 3

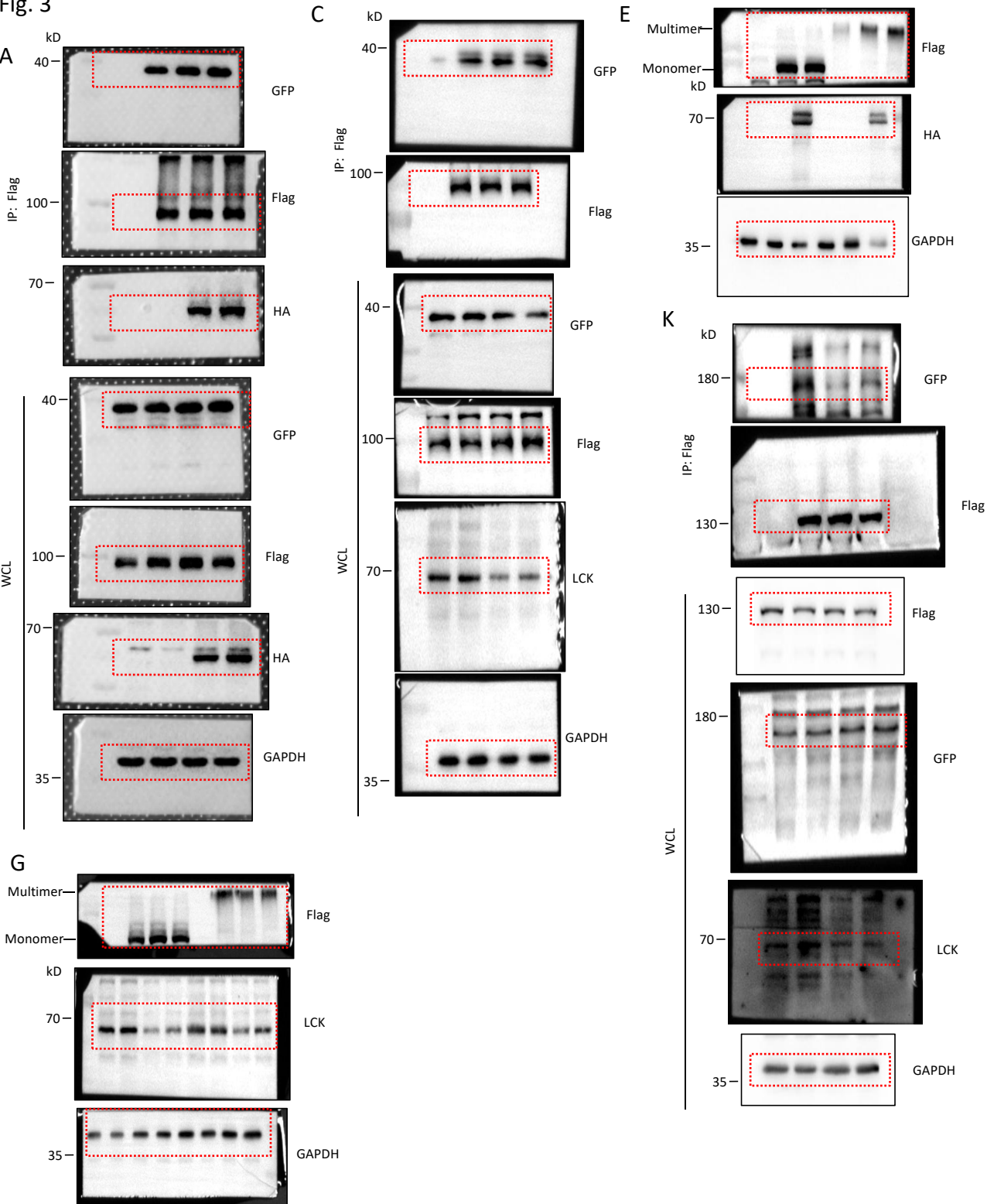

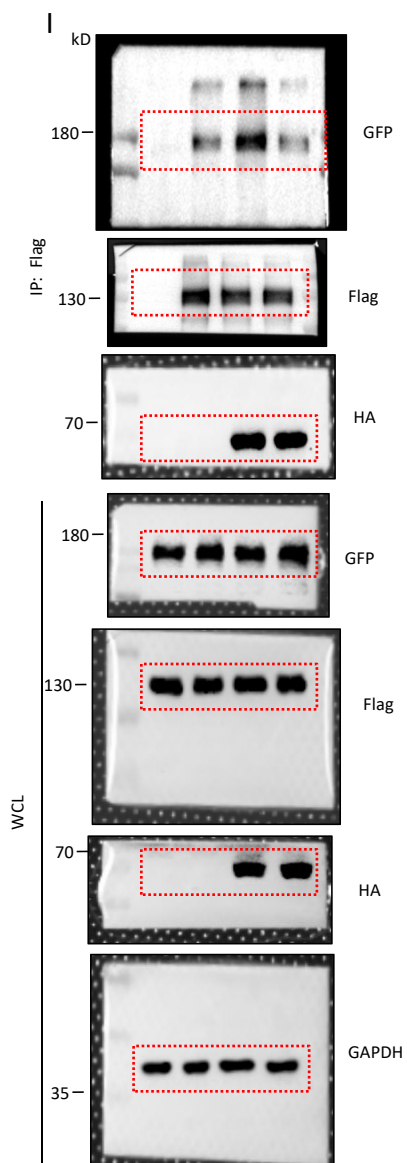

Fig. 4

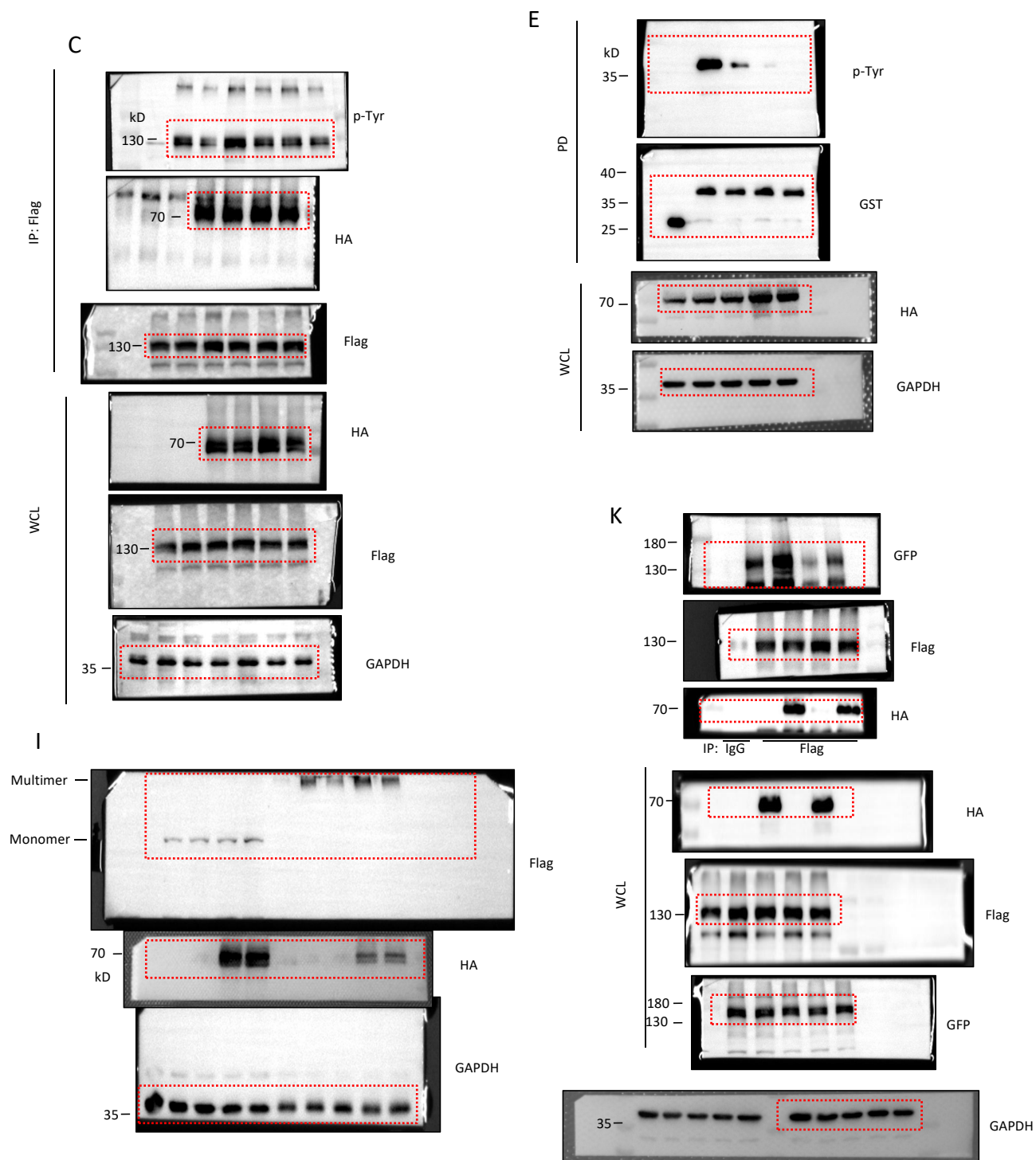

Fig. 5

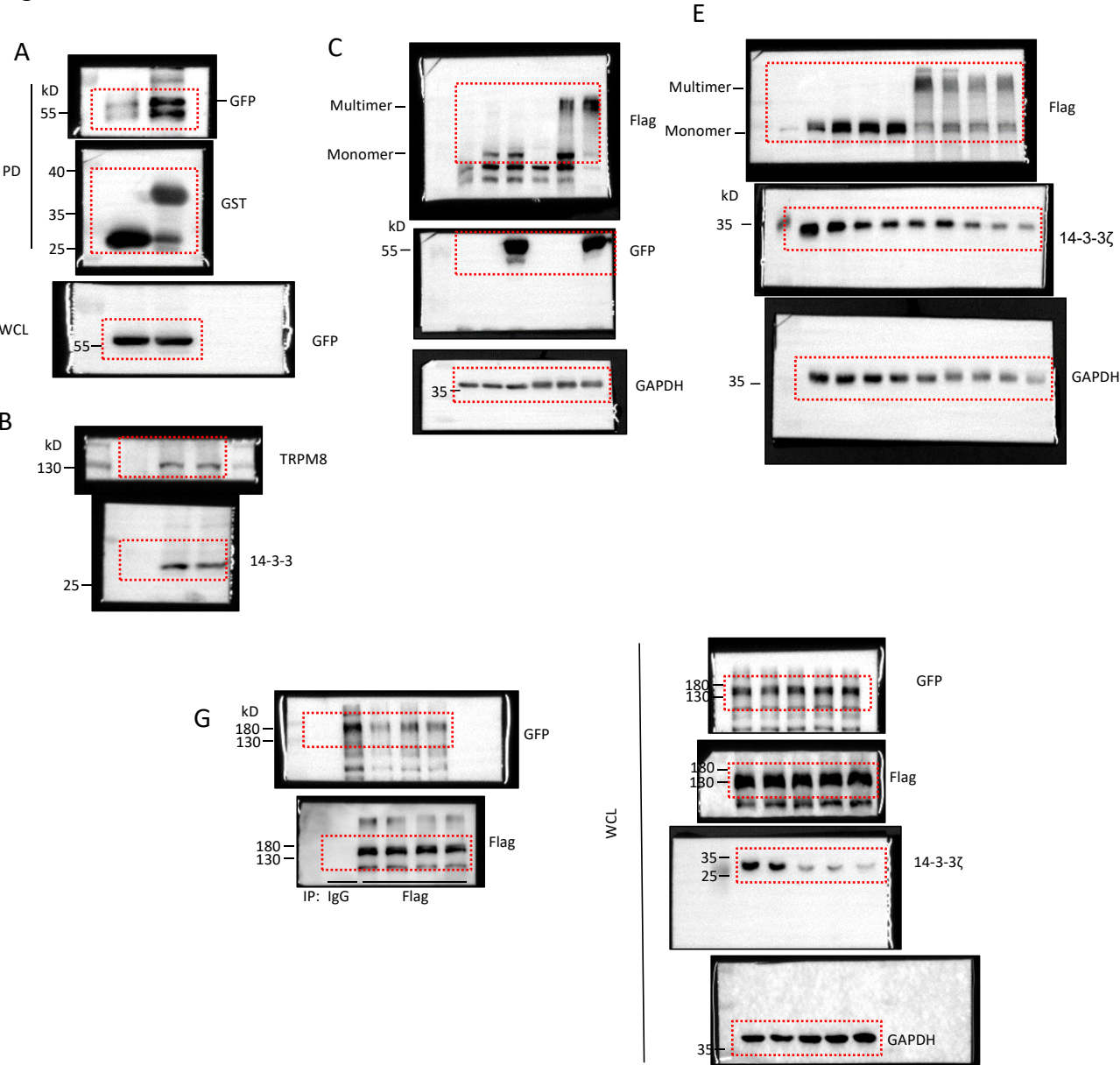

Fig. 6

A

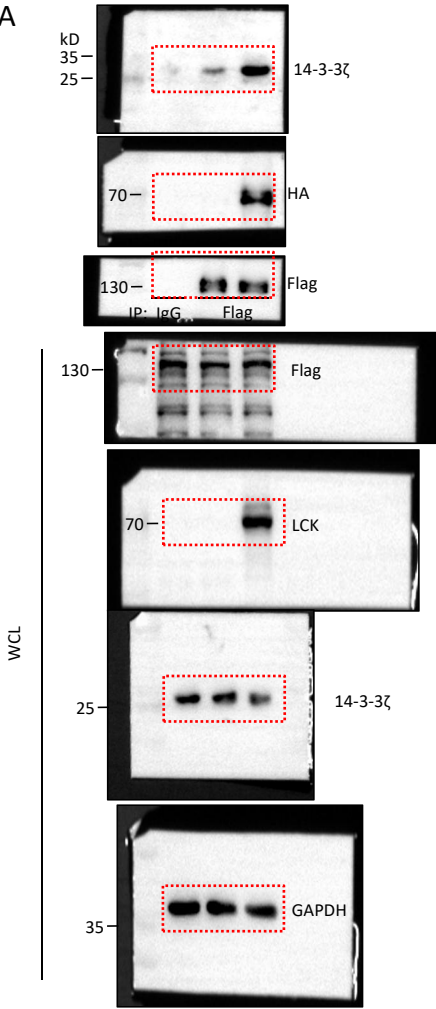

C

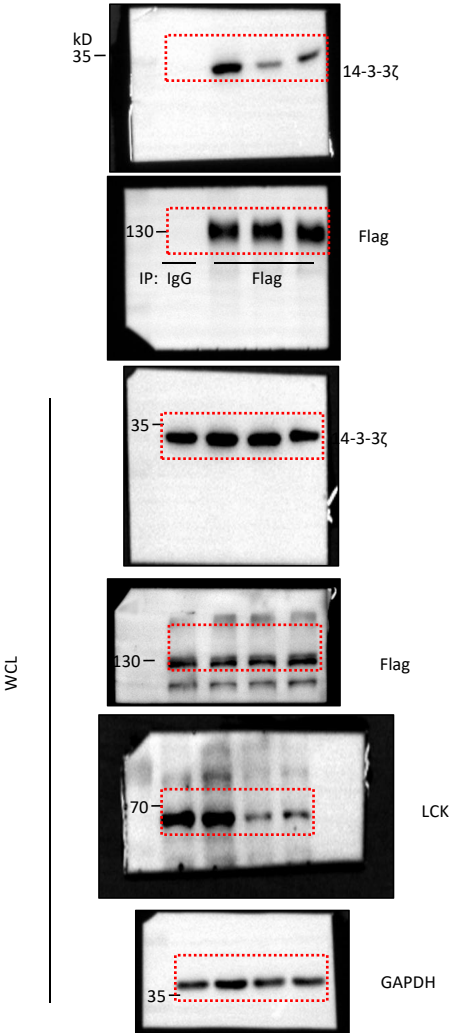

E

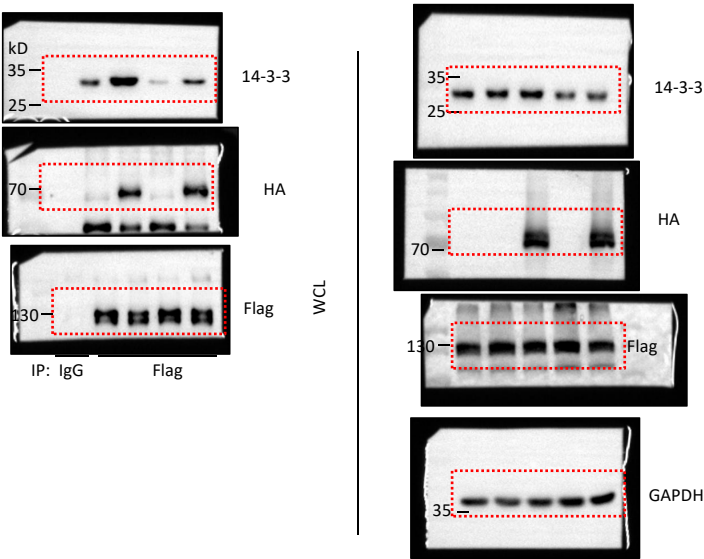

Fig. 6

G

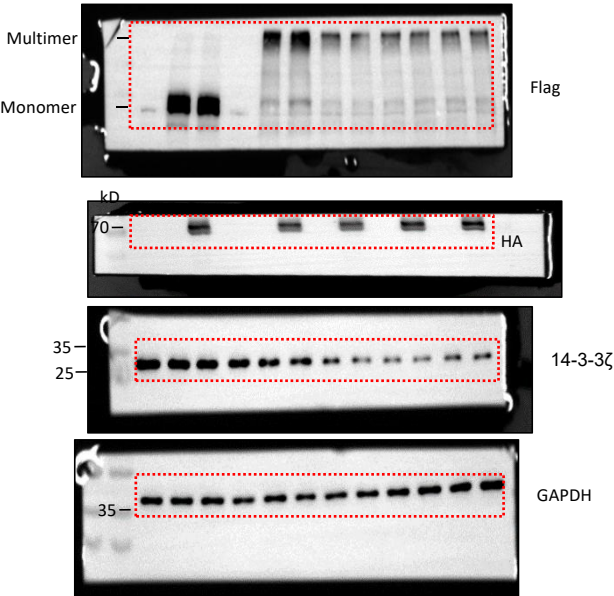

I

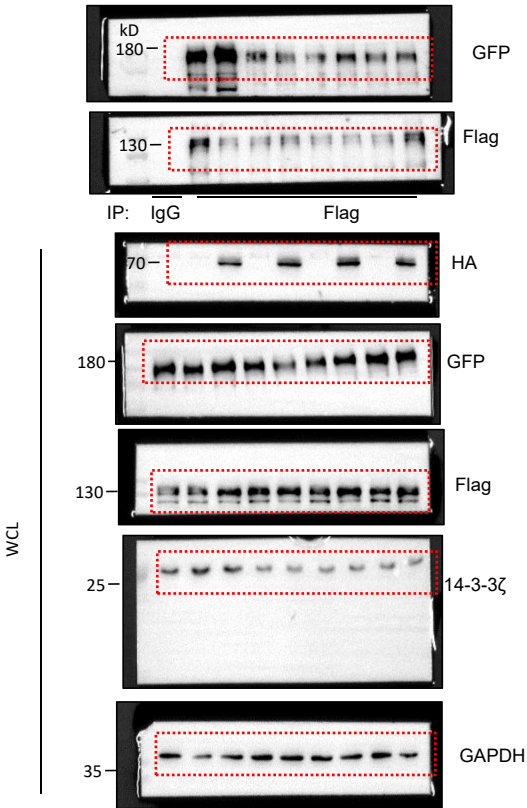

Fig. 7

A

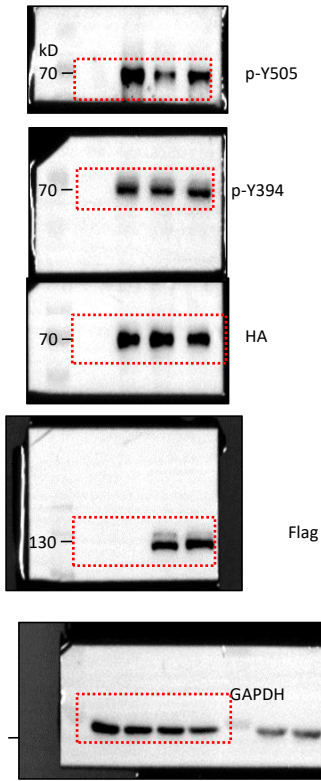

C

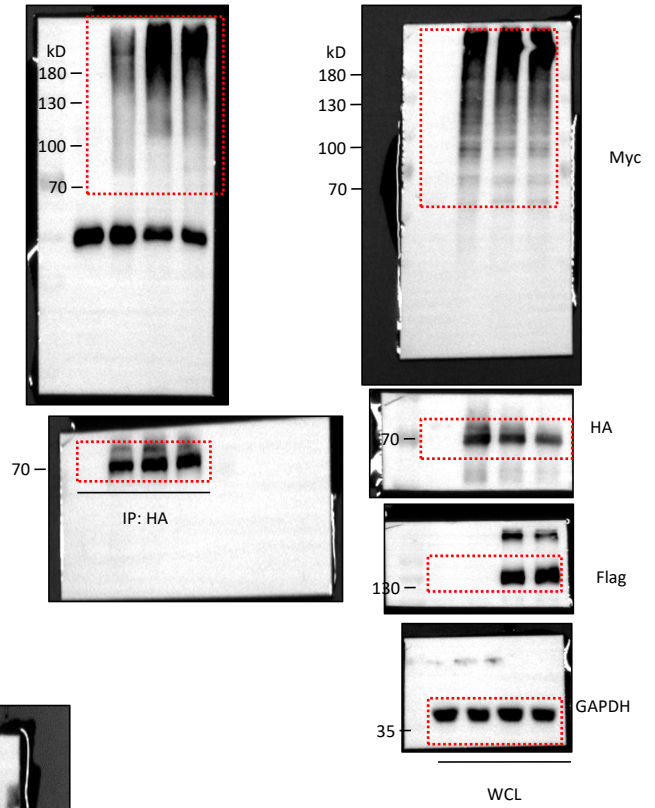

E

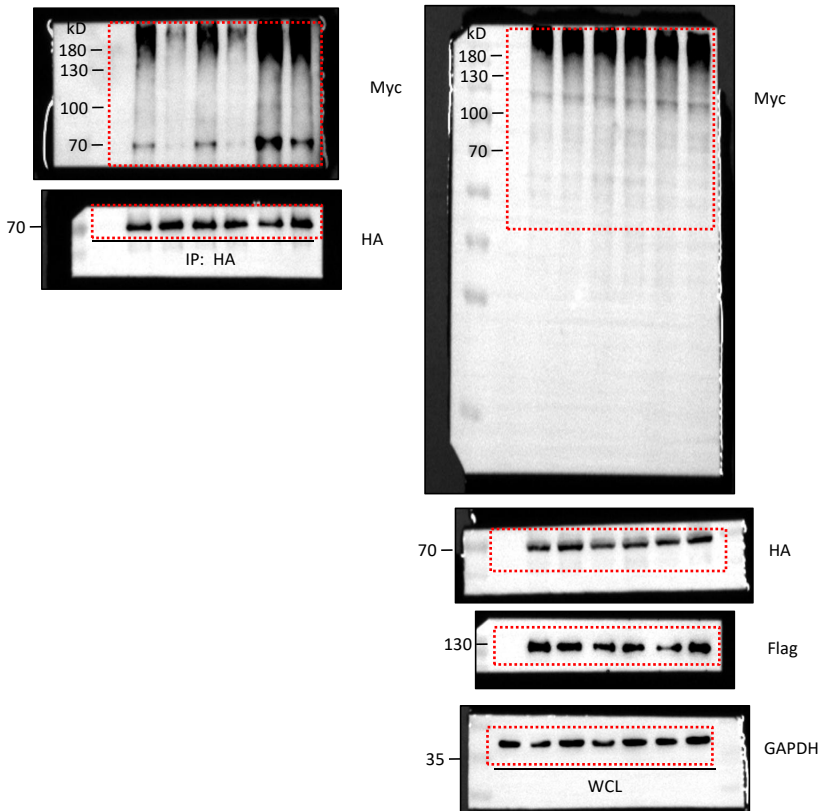

Fig. S1

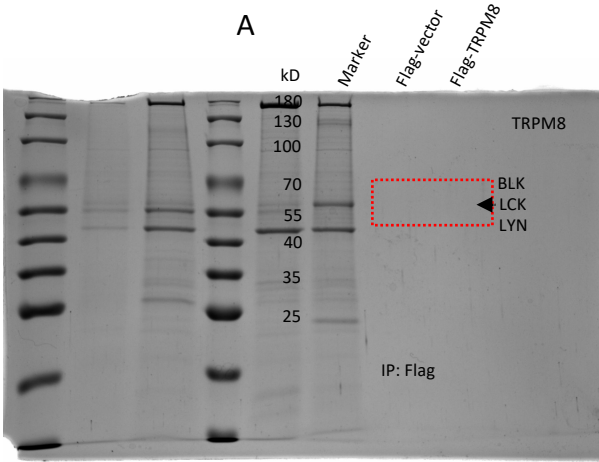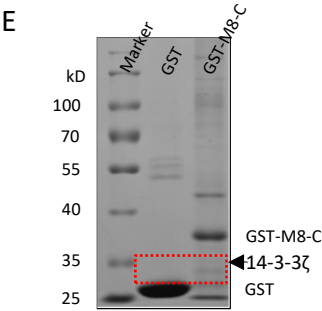

Fig. S2

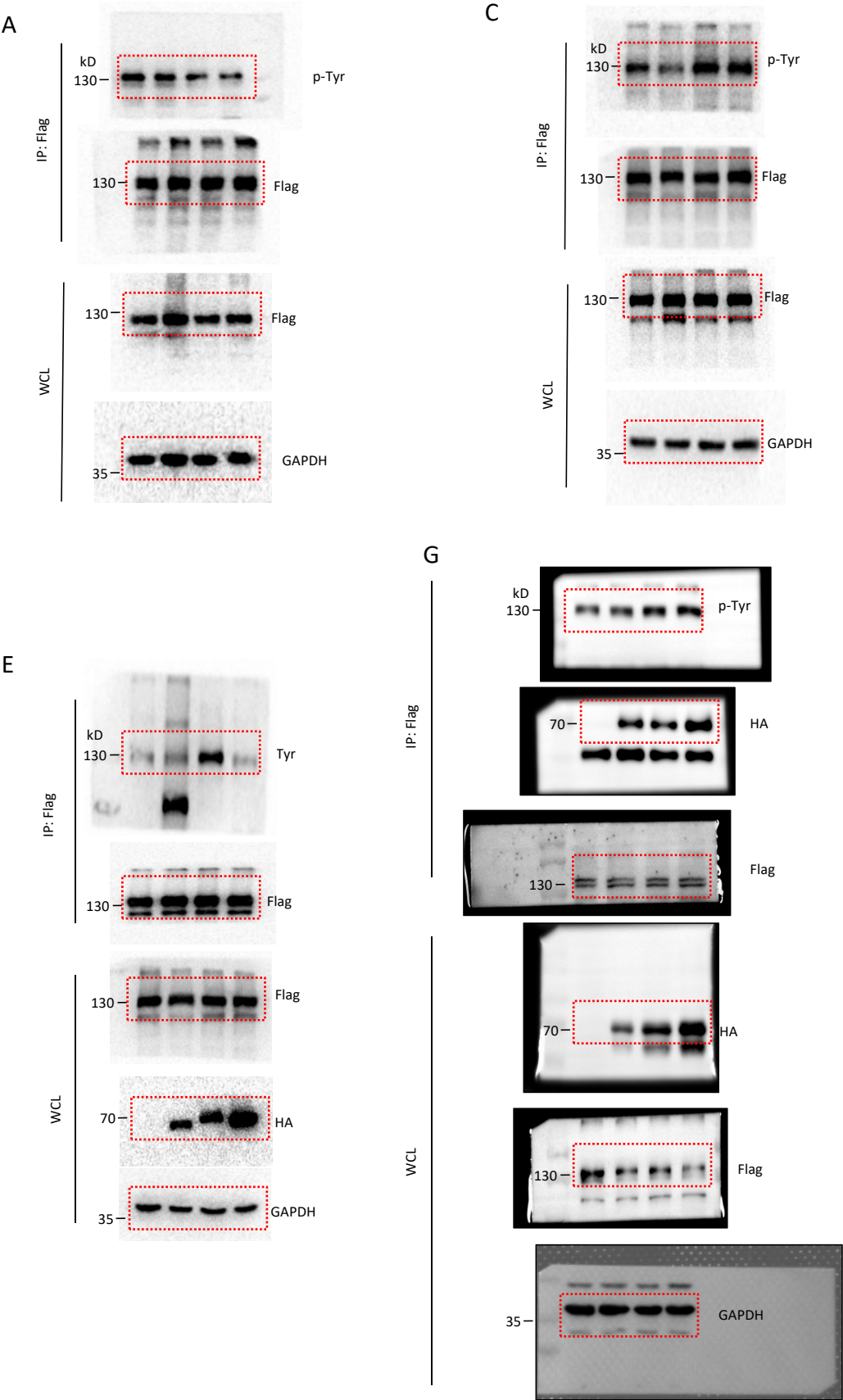

Fig. S2

K

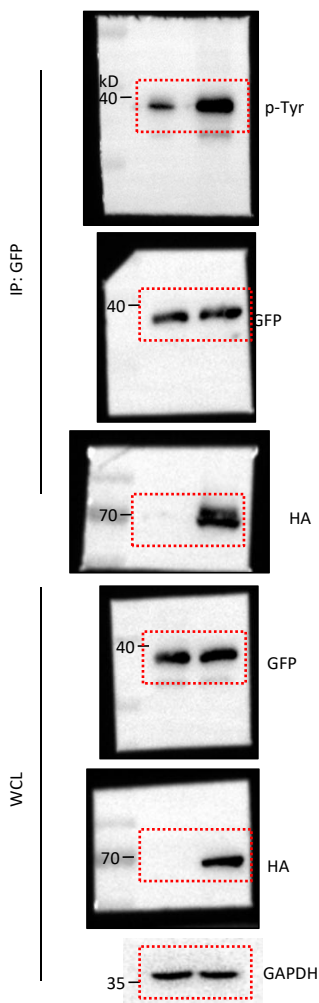

I

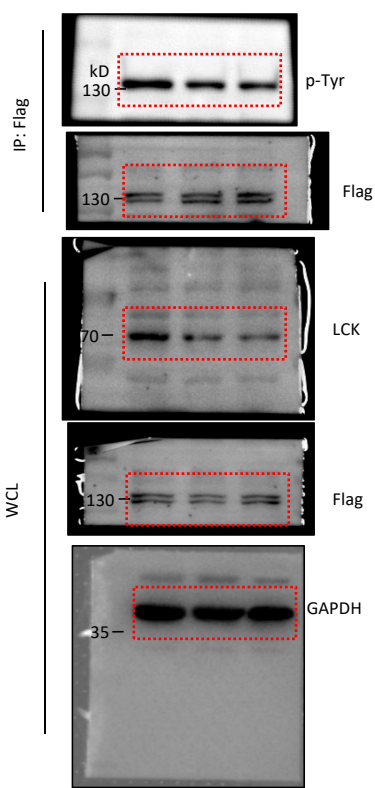

M

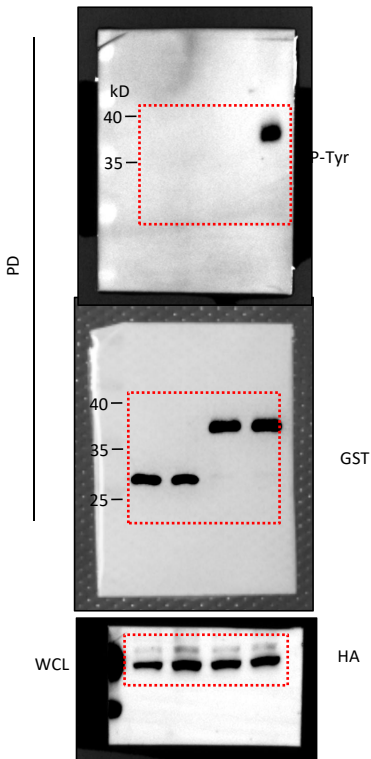

Fig. S3

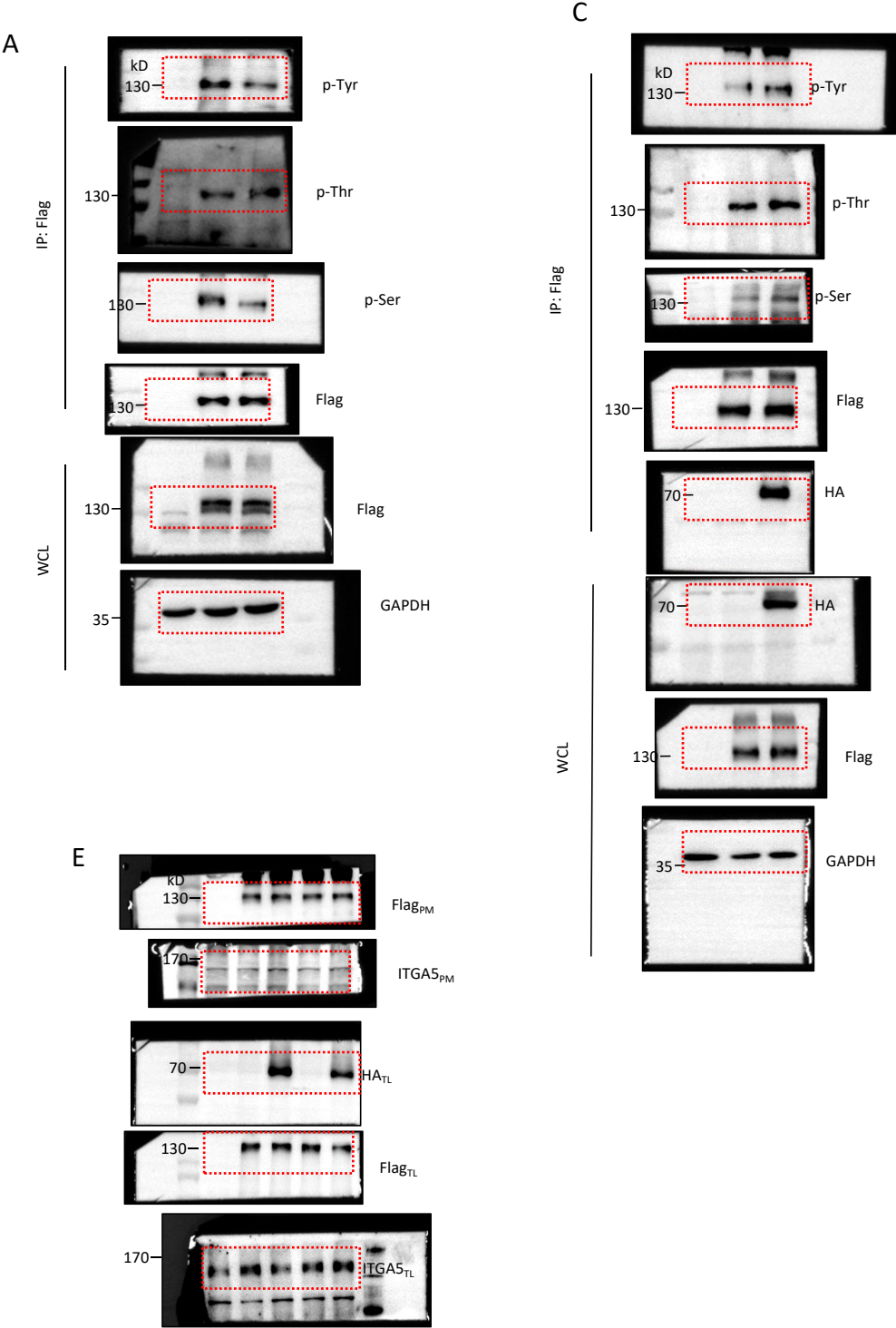

Fig. S4

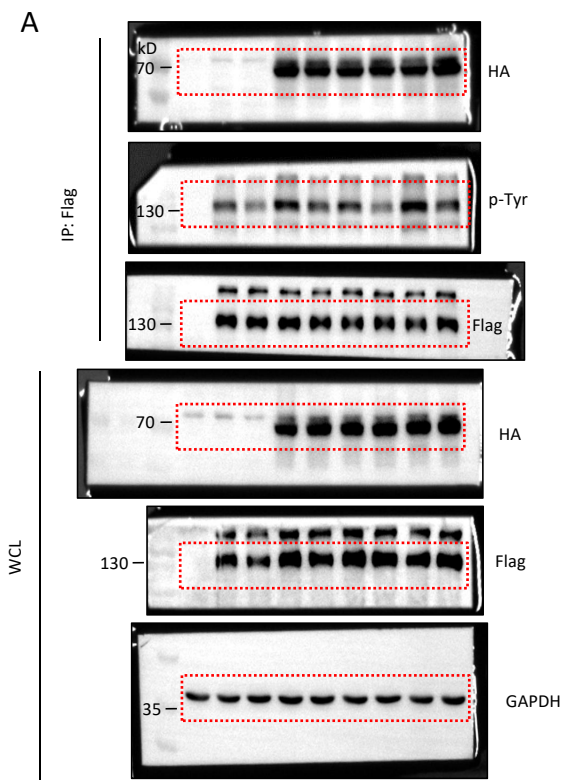

Fig. S5

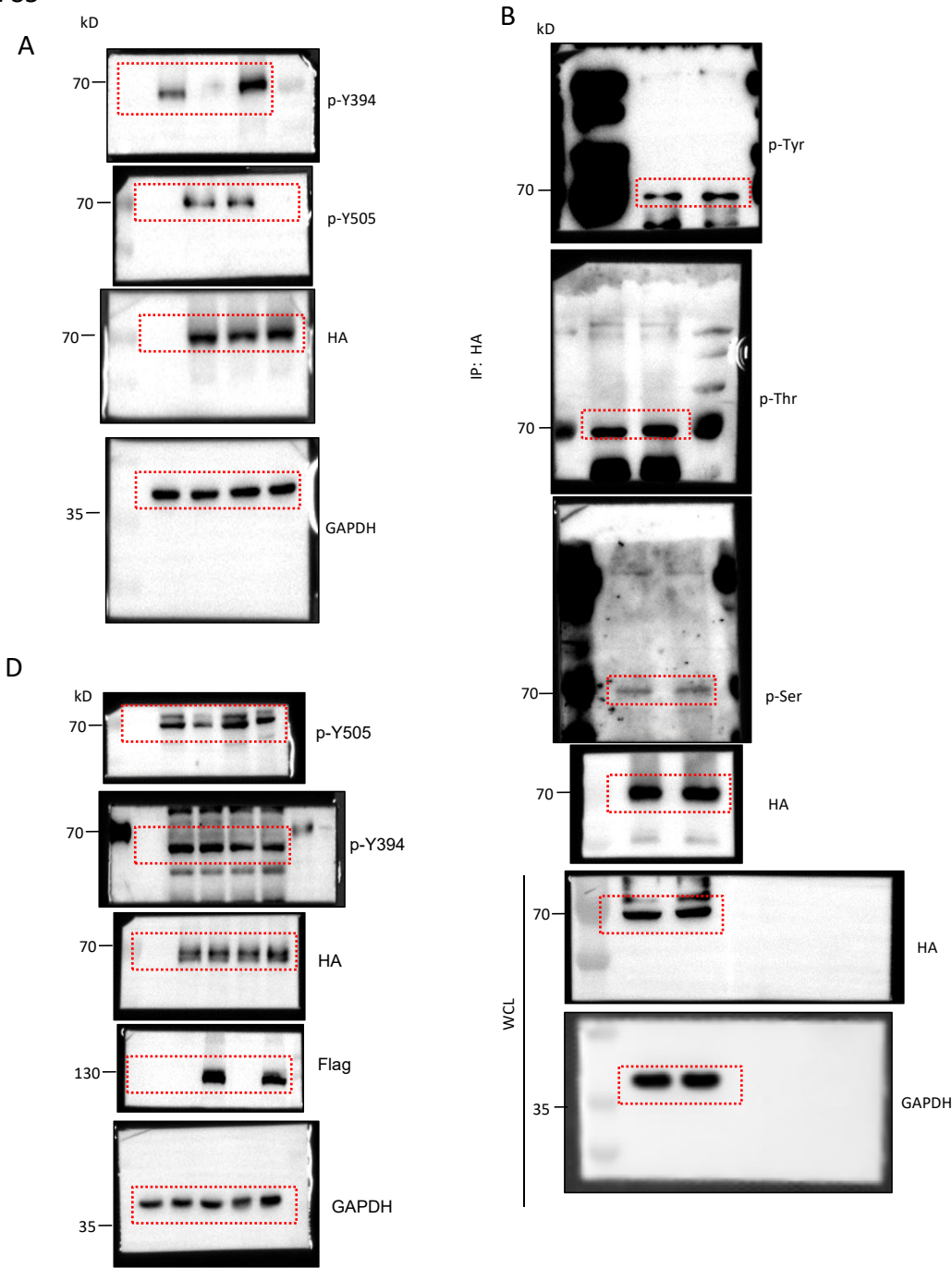

Supplement: Supplementary file 2 — Full length uncropped original western [file 41419_2022_4977_MOESM2_ESM.pdf]
